# Doxorubicin-NFL-TBS.40-63 peptide Gold Complex Nanovector (DOX IN-NFL@AuNPs): Efficacy Evaluation in a mouse transplantation tumor model induced by PANC-1/ADR human pancreatic cancer resistant strain cells

**DOI:** 10.7150/ntno.109280

**Published:** 2025-06-19

**Authors:** Hui Liu, Qiqian Liu, Xiaowu Li, Abdelkader Boucetta, Joel Eyer, Jolanda Spadavecchia

**Affiliations:** 1 Department of Hepatobiliary Surgery, Guangdong Provincial Key Laboratory of Regional Immunity and Diseases & Carson International Cancer Center, Shenzhen University General Hospital & Shenzhen University Clinical Medical Academy Center, Shenzhen University, Shenzhen, 518083 China.; 2 CNRS, UMR 7244, NBD-CSPBAT, Laboratoire de Chimie, Structures et Propriétés de Biomatériaux et d'Agents Thérapeutiques Université Paris 13, Sorbonne Paris Nord, Bobigny 93000, France; University Sorbonne Paris Nord; CB3S, UMR CNRS 7244, Insitut Galilée, 99 France.; 3 Laboratoire Micro et Nanomédecines Translationnelles, Inserm 1066, CNRS 6021, Institut de Recherche en Ingénierie de la Santé, Bâtiment IBS Institut de Biologie de la Santé, Université d'Angers, Centre Hospitalier Universitaire, Angers, 49100 France.

**Keywords:** BIOT-NFL peptide, Doxorubicin, Gold Complex, PDAC, immune system.

## Abstract

The key role of the NFL-TBS.40-63 peptide (BIOT-NFL) is to target and destroy glioma cancer cells. Recently we have performed a novel peptide-hybrid-gold nanovector (BIOT-NFL-PEG-AuNPs) capable to destroy microtubule network of pancreatic cancer cells (PDAC) exhibiting a decrease of tumor index with a real anti-angiogenic effect. In order to improve the scientific background of our study, we conceived a chemotherapeutic hybrid nanovector based on gold-doxorubicin (DOX) functionalized with the NFL-TBS.40-63 peptide (BIOT-NFL) as a promising therapeutic in PDAC cancer. Mouse transplantation tumor model induced by PANC-1/ADR human pancreatic cancer resistant strain cells, was used to evaluated the therapeutic efficacy of DOX IN-NFL@AuNPs as chemotherapeutic nano-drug. Our results indicate that DOX IN-NFL@AuNPs have a great impact on the decrease of the tumor growth and decreased the tumor index with a relevant effect on cytokines and ROS levels, thus confirming the impact of DOX IN-NFL@AuNPs to boost the immune system.

## Introduction

Pancreatic Ductal Adeno Carcinoma (PDAC) has one of the highest mortality rates of any cancers^1^. No efficient treatments have been found for improving the prognosis and reducing the deaths of patients due to PDAC. Typically for the late-stage diagnoses limit successful surgical resections, with 80-85% being unresectable at the time of diagnosis^2^. The alternative therapies could only be relied on the chemotherapeutics such as doxorubicin, gemcitabine and paclitaxel^3^,^4^,^5^, nevertheless, the difficulty for chemotherapy treatment of PDAC specially link to the presence of a dense desmoplastic reaction (DR)^6^ which consists a largely portion of fibroblasts, pancreatic stellate cells (PSCs), and extracellular matrix (ECM) proteins, including collagens I and III and fibronectin^7^,^8^*.* In another hand, the stroma^9^,^10^ includes endothelial cells, immune cells, pericytes, and nerve fibers. All these factors create a solid barrier preventing the conventional chemotherapeutics penetrate inside the PDAC (chemo-resistance). Some of the widely used chemotherapeutics, as doxorubicin, have been suggested to induce anti-tumor immunity through the stimulation of immunogenic cell death (ICD)^11^. Recent research shows that the cytotoxic chemotherapy induces the generation of anti-cancer immunity and durable tumor responses, which is considered as a promising method to treat cancer 12-14.

To increase the durability of anti-cancer immune responses, both antigen recognition and adjuvant signals result from cell stress or death were required^15^,^16^. Immune-stimulating chemotherapies incite the release of pro-inflammatory signals, including damage-associated molecular patterns (DAMPs), that indicate danger and act as immunologic adjuvants, provoking anti-tumor immunity. However, even in settings where tumor antigens are present, cytotoxic chemotherapy rarely generates durable anti-cancer immune responses. This suggests that any immune stimulus from genotoxic therapy is insufficient or ultimately suppressed^62^. To overcome the drug resistance, lasting the durability of cytotoxic chemotherapy in terms of antigen recognition and adjuvant signals for PDAC treatment, it is urgent to find some new alternatives, hence drug delivery systems (DDSs) could be considered as an efficacious system^17^. Different nanocarriers such as dendrimers^18^,^19^, liposomes^20^, metal nanoparticles^21^, polymer micelles and vesicles^22^,^23^ have been utilized to improve the targeted delivery of the therapeutics for cancer treatment.

Among all the DDSs,the application of gold nanoparticles (AuNPs) is growing field^24^,^25^. The inherent, non-toxic and biocompatible properties make them a promising vehicle for drug delivery. Therefore the biomolecule-conjugated gold nanoparticles are considered as the promising candidate in our studies due to its surface properties which can be tuned by using biomolecules, such as, antibodies and peptides^26^. The NFL-TBS.40-63 peptide has been conceived by J. Eyer and his team 31-33. This peptide interacts specifically in several glioblastoma cell lines reducing glioblastoma viability through the blood-brain barrier inducing the inhibition of the formation of microtubules^34^. NFL-TBS.40-63 peptide is also internalized massively in glioblastoma cells and poorly in other cells from the nervous system, like astrocytes and neurons. Recently, we have compared the power of NFL-TBS.40-63 peptide onto pancreatic cancer cells; for this aim we synthetized a gold-complex biotinylated NFL-TBS.40-63 (BIOT-NFL) to form a hybrid gold nanovector (BIOT-NFL-PEG-AuNPs), by methodology IN discovered by J. Spadavecchia et al.^27^-^30^.

It was demonstrated for the first time that BIOT-NFL-PEG-AuNPs have the ability to target the destruction of pancreatic cancer cells (PDAC) under *in vivo* experimental conditions, by varying the metabolic profiles of these MIA-PACA-2 cells^27^.

It is known that the PDAC response to stiffness and solid stress in pancreas, is driven by the change in ECM-Cell interaction, influenced mainly by the behavior of the micro-fluidic conditions. With those new controlled peptide structure and conditions, we are presenting simultaneously in the future study, a new multi-physics concept to base a proof of concept and hypothesis of computational analysis in pancreatic complex network, enhancing the reverse impact of the PDAC on the ECM, and controlling the micro-fluidic conditions change to promote peptides pathways and strength, with a customized potential. By generating a non-favorable ECM platform for tumor growth, a biomechanics effects destroys molecular messengers and Tumor growth factor, like β (TGF-β) or Sonic hedgehog (SHH) altering ECM deposition by cancer-associated fibroblasts (CAFs), compromising the pro-tumorigenic programs.

Encouraged by our previous results, we have decided to integrate chemotherapeutic doxorubicin in nanocomplex to formulate a new advanced nanoformulation (DOX IN-NFL@ AuNPs). To estimate the therapeutic efficacy of DOX IN-NFL@AuNPs, a mouse transplantation tumor model induced by PANC-1/ADR Human Pancreatic Cancer Resistant Strain Cells was applied. Several *in vivo* studies included different concentrations of DOX and BIOT-NFL into nanovectors have been carried out and the relevant results show that DOX IN-NFL@ AuNPs have a high impacted on the tumor growth and consequently decreased the tumor index without change body weight of mice. An excellent induction of ROS reaction, confirms the ability of DOX IN-NFL@ AuNPs to alter the metabolic profiles of PANC-1/ADR Human Pancreatic Cancer Resistant Strain Cells. The cytokine levels were also detected to evaluate the effect of serum inflammatory factors and the power of DOX IN-NFL@ AuNPs to boost the immune system.

We assume that this study is very important to provide a newly advanced PDAC peptide-chemotherapeutic therapy which could overcome the limits and poor effects of the classic chemotherapeutic drugs for PDAC therapy due to drug resistance following desmoplastic stroma. In the further study, as initiated previously, the new concept of micro-fluidic potential spread, driven by the change of ECM-Cell micro-environment and stiffness, will leverage the capability and the nutritional factors of the tumor progress. One field of application of this new hypothesis computational model including the new peptides described in this study, will permit to control the growth of the metastases process. Having a growth tumor endothelial remodeling, driven by tumor migrating compounds (TMC) like CTC and EV**'**s expanding the metastases. Combine the provided numerical output data, with the controlled structure, the new nano-formulation and the physical conditions of the DOX IN-NFL@AuNPs, permit to well customize the injection rate locations and pathways, and target the reinforcement spread of the electrical potential by adapting its Zeta potential, in order to build a specified ECM and virtual layers of Basements Membrane (BM) around the tumor cellular matrix. The aim is to maintain a targeted environment surrounding initiated PDAC deposition, promoting tumor vessel collapse and osmolarity hijacking. In other hand, the new model and concept will support the peptides to use the ECM and BM compounds and tissue matrix organization, to control the environment physical condition by generating a targeted spinning flow, changing micro-fluidic viscosity and shear stress. This configuration creates a sever micro-environment with thresholds well above the resistance of the TMC membrane, hence, destroying and disrupting metastatic pockets.

## Methodology

### Chemical design and conception of gold nanovector (DOX IN-NFL@AuNPs)

J. Spadavecchia *et al*. have exhaustively studied the chemical mechanism of complexation methodology (Method IN) with a large library of drugs, biomolecules (proteins, enzymes, aptamers, biological cofactors) as stabilizers and/or reagents on the hybrid- nanoparticles^30^-^35^. Recently, the same authors have designed and protected, the encapsulation of biotinylated cell penetrating peptide (CPP) as hybrid theragnostic complexes and their biological activity (P7391FR00-50481 LIV)^27 35 36^. In the present study, we developed a novel hybrid gold nanovector in which biotinylated-NFL and DOX participate actively in the grow process to obtain hybrid gold nanovectors (DOX IN-NFL@AuNPs) chemically stable and biological efficient.

**Scheme 1:**
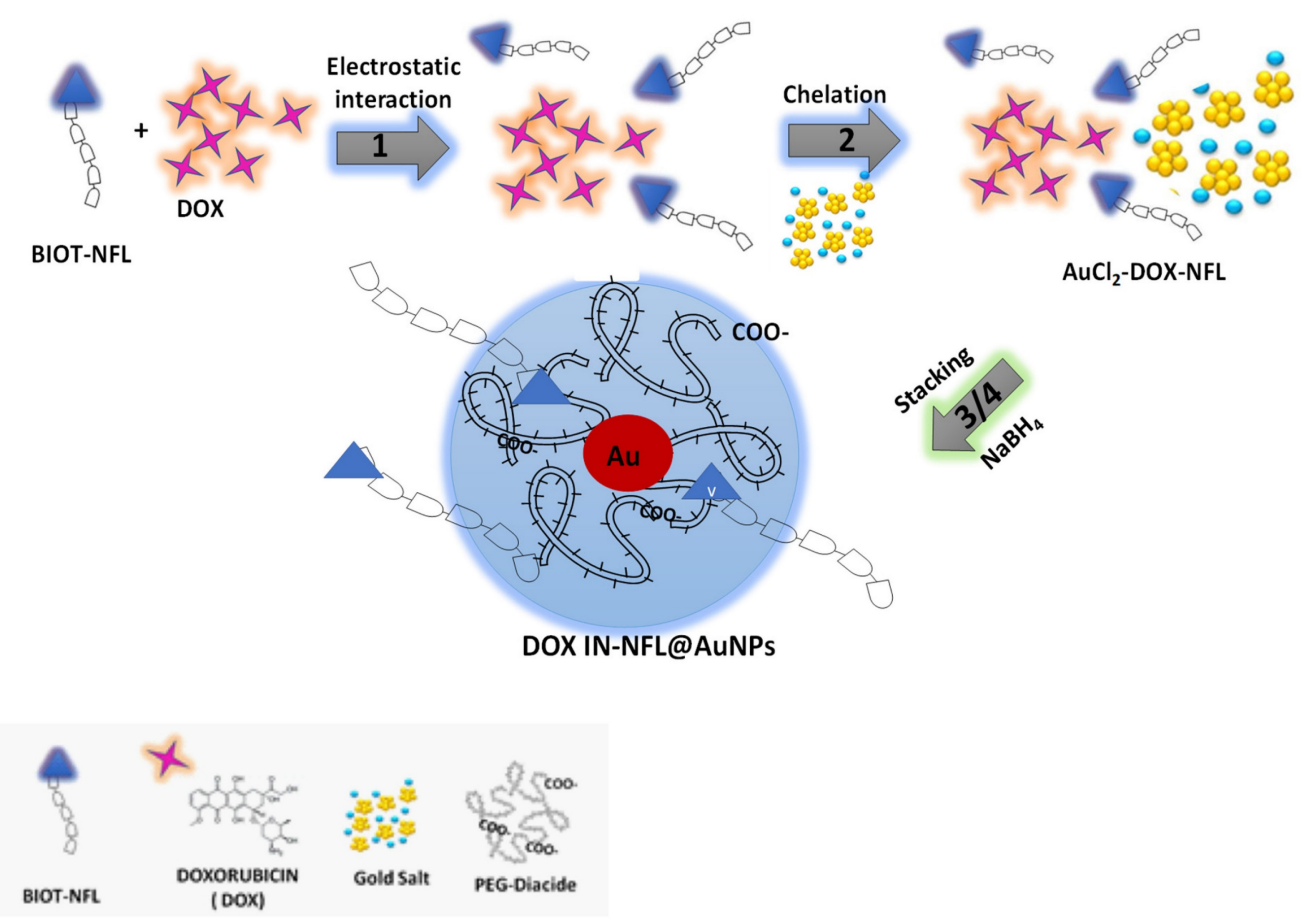
Schematic design of the chemical methodology to obtain DOX IN-NFL@AuNPs (Method IN).

The formation of DOX IN-NFL@AuNPs comprehends the chemical steps depicted in **Scheme 1**:

(1) Electrostatic interaction between biotinylated peptide NFL-TBS-60-43 (BIOT-NFL) and Doxorubicin (DOX) to generate hybrid-DOX-peptide (DOX-NFL),

(2) Chelation between DOX -NFL complex and gold salt (AuCl_4_^-^) to form hybrid-DOX-peptide (DOX-NFL) gold cluster^28 30^ (AuCl_2_-DOX-NFL),

(3) Stacking process of biocompatible polymer (PEG-Diacide) onto hybrid-DOX-peptide (DOX-NFL) gold cluster (AuCl_2_-DOX-NFL),

(4) Complete reduction and formation of hybrid-gold nanovector (DOX IN-NFL@AuNPs).

In the first step, the biotinylated peptide NFL-TBS-60-43 (BIOT-NFL) and doxorubicin (DOX) were mixed and interacts through the positively charged amino (-NH_3_^+^) of DOX and carboxylic group (COO^-^) of peptide in water solution, producing an electrostatic complex (DOX-NFL). In the second step, the obtained DOX-NFL interacts with a gold salt solution (HAuCl_4_), thanks to the chelation between hydroquinone-quinone and gold as discussed previously^28 30^. The further addiction of the polymer (PEG diacide) onto hybrid-DOX-peptide (DOX-NFL) gold cluster (AuCl_2_-DOX-NFL), promotes the kinetics of reduction by chelation with the Au ions^21^ and the colloidal stabilization after reduction process in the final step.

## Results and Discussion

### Spectroscopic Evaluation

**Figure 1:**
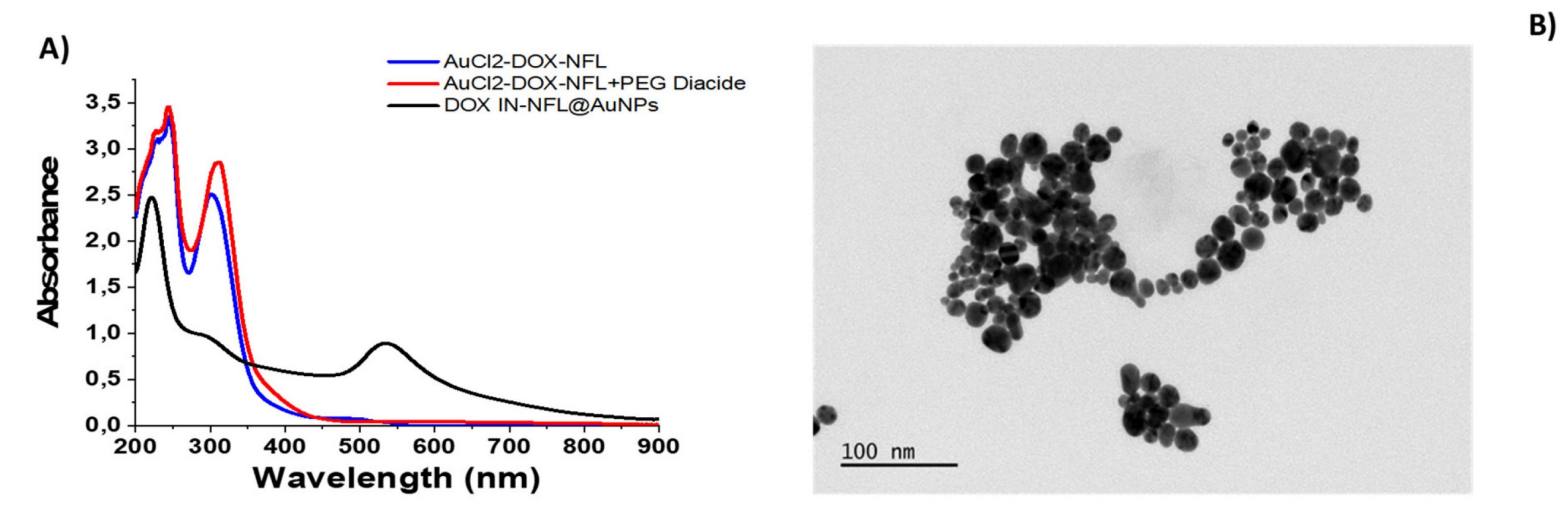
A) UV-Vis absorption of DOX IN-NFL@AuNPs (black line); each step of gold complex formation was carried out: AuCl2- DOX-NFL (blue line); DOX-NFL-AuCl2-PEG Diacide (red line); B) TEM images of DOX IN-NFL@AuNPs. Scale bars: 100 nm.

As followed in **Figure 1A, black line**, the UV-Vis spectra of the DOX IN-NFL@AuNPs, exhibits a small peak at 295 nm and a characteristic peak at 215 nm responsible to π-π* electronic transitions associated to peptide's backbone.

The electrostatic interaction between BIOT-NFL-peptide and DOX followed by complexation to gold salt to form DOX-NFL-AuCl_2_^-^, display two peaks at 230-242 nm and a characteristic peak at 312 nm due to gold salt complex (**Figure 1-A, blue line**). After polymer stacking step (AuCl_2_-DOX-NFL-PEG diacide) we observed a strong decrease of the peak at 312 nm and the peak at 218 nm (**Figure 1A, red line**) with appearance of small peak at 480 nm 30 due to the formation of gold clusters ascribed to reduction started by carboxylic groups of PEG^30^. This spectroscopic fingerprint was associated to π-π* electronic transitions between the NFL-peptide backbone and gold salt ions, with a variation of the steric arrangement of peptide under gold-salt complex and migration through PEG molecules as discussed previously^36^. Finally, the reduction mixture with NaBH_4_ reduces completely gold species from Au^III^ to Au^0^ to form DOX IN-NFL@AuNPs (**Figure 1A, black line**).

TEM images of DOX IN -NFL@AuNPs display a polydisperse nanoparticles with an average size of 15.52 ± 4.4 nm obtained by Image J software (Particle Analysis (imagej.net); ImageJ) onto each Image TEM (**Figure 1-B**). The hydrodynamic diameter measured by DLS technique is about 85 ± 2 nm due to steric arrangement of polymer onto gold peptide complex with a Zeta potential of -35.7 mV (**data showed in Supporting information**). As proved previously, the presence of biotin onto peptide improves a better chemical and steric configuration and consequently active targeting on cancer cells.

### *In vivo* antitumor efficacy

Biomolecules targeting microtubules are extensively applied in cancer therapy with a good efficacy^38^. However, their administration provokes several secondary effects. Thanks to the advancement of research and knowledge on the structure of tubulin, a better comprehension of the mechanism of action provided the development of original therapeutic approaches^39^.

Doxorubicin (DOX) is an anthracycline with a chemical structure well defined and studied with high activity in cancer therapy^40^. The major issue has been the Doxorubicin (DOX), conferring drug resistance^41^,^42^. Whereas DOX-conjugated NPs have been used as drug carrier and delivery platform especially in treating several human cancers^43^,^44^, their use in the treatment of human pancreatic cancer cells has been little explored. Since 2016 Spadavecchia et al. promoted a novel methodology in which drugs and/or biomolecules were chelated to gold salt to form hybrid nanovector. In this steric and chemical conformation, we obtained excellent results in terms of efficacy *in vitro* and *in vivo*^28,30,45,46.^

As discussed previously, J. Eyer and co. have analyzed a peptide which sequence on the neurofilament light subunit (NFL-TBS.40-63) is capable to fix tubulin dimers on specific sites^13^ and can inhibit the proliferation of glioma cells by destroying their microtubule network^38^.

**Figure 2:**
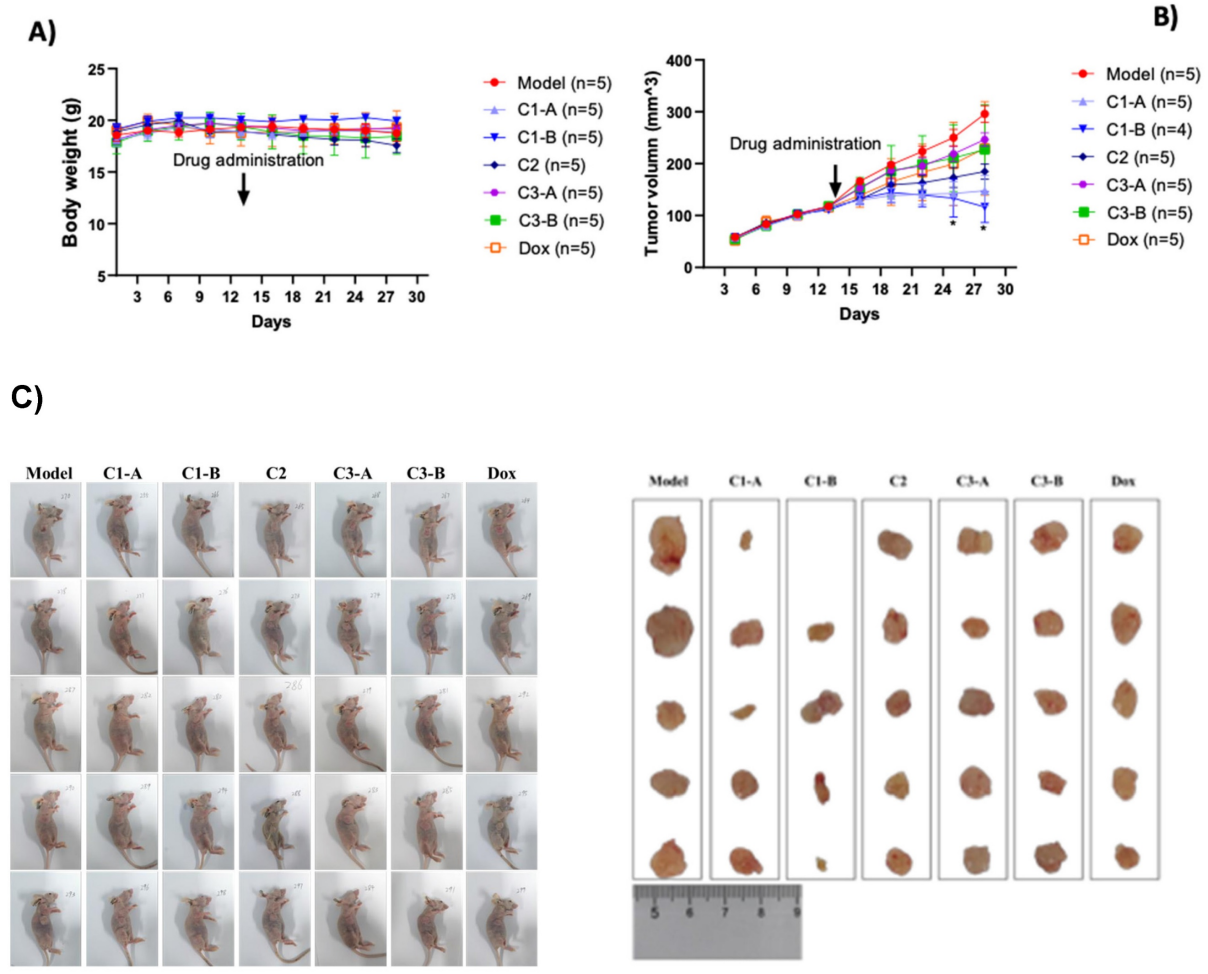
A) Effect of DOX IN-NFL@AuNPs on the body weight of pancreatic cancer in mice. Body weight was shown as Mean ± SD by Two-way ANOVA. Model group compared with DOX IN-NFL@AuNPs group, *p<0.05 and **p<0.05. B) DOX IN-NFL@AuNPs on the tumor volume of pancreatic cancer in mice. Tumor volume (mm3). C) Representation of tumor volume sizes of tumor-bearing nude mice in each group.

Recently, we realized a gold nano formulation composed by biotinylated-NFL-TBS.40-63 peptide (BIOT-NFL) complexed to gold salt and pegylated chains (BIOT-NFL-PEG-AuNPs) and we have studied their power of internalization on PDAC cells (P7391FR00-50481 LIV) including the capacity of the BIOT-NFL-PEG-AuNPs to target mouse transplantation tumor model PDAC improving their anti-cancer efficacity^38^. Herein we study the dual therapeutic effect of DOX as chemotherapeutic agent and NFL-TBS.40-63 peptide (BIOT-NFL) as gold nanoformulation depicted in **Table 1**, under some experimental conditions^38^. The therapeutic effect of DOX IN-NFL@AuNPs was monitored using mouse transplantation tumor model under hypodermic injection as shown in **Figure 2A**. All nanomaterials (C1-A-C3-B) did not significantly affect the body weight of PANC-1/ADR ruffed nude mice. We also observed a reduction of tumor (**Figure 2B**). The results of white light plots, tumor volume curves and endpoint tumor volume sizes of tumor-bearing nude mice in each group are shown in **Figure 2B** -**2C**. Indeed, at the end of the experiment, the nude mice in each group were dissected and the tumor and each organ were weighed to calculate the tumor and organ indices (**Figure S1 in Supporting Information**). As shown in **Figure S1 in Supporting Information**, C1-A and C1-B could reduce the tumor indices, and there was not significant difference in the organ indices of the remaining groups.

C1-B more significantly reduced the tumor volume of pancreatic cancer model mice (p < 0.05) (**Figure 2B**). On the 28th and 31st days and meanwhile the model group (PANC-1/ADR) significantly reduced the tumor size of pancreatic cancer mice as well (p < 0.05).

The presence of DOX in gold nanoparticles confers a different steric stability of the peptide allowing the destruction of microtubules.

### ROS evaluation

It was established that doxorubicin-induced ROS over production occurs inside mitochondria and is mediated by the mitochondrial NADPH oxidase activity^47^. Previously we compared the effect of free DOX, before and after encapsulation in polymer gold complex^28 30^. Following this methodology^30^, DOX was chelated to gold salt previous deprotonation of C24 before interaction to polymers chains. In this steric conformation, DOX chelated to gold complex do not bind Fe^3+^/Fe^2+^ and so do not promote ROS production responsible of cardiotoxicity. Previously, we evaluated the biological effect of the NFL-TBS 60-43 peptide on glioblastoma and pancreatic cell mitochondria48. Others authors indicated that NFL can interact directly with mitochondria, such as vimentin, to regulate mitochondrial motility interacting with a N-terminal domain of vimentin^49^. Based on previously studies, we have demonstrated the exceptional activity of a combined formulation composed by NFL-TBS.40-63 peptide under biotinylated form and doxorubicin into gold complex nanoparticles (**DOX IN-NFL@AuNPs**) to PDAC.

**Figure 3:**
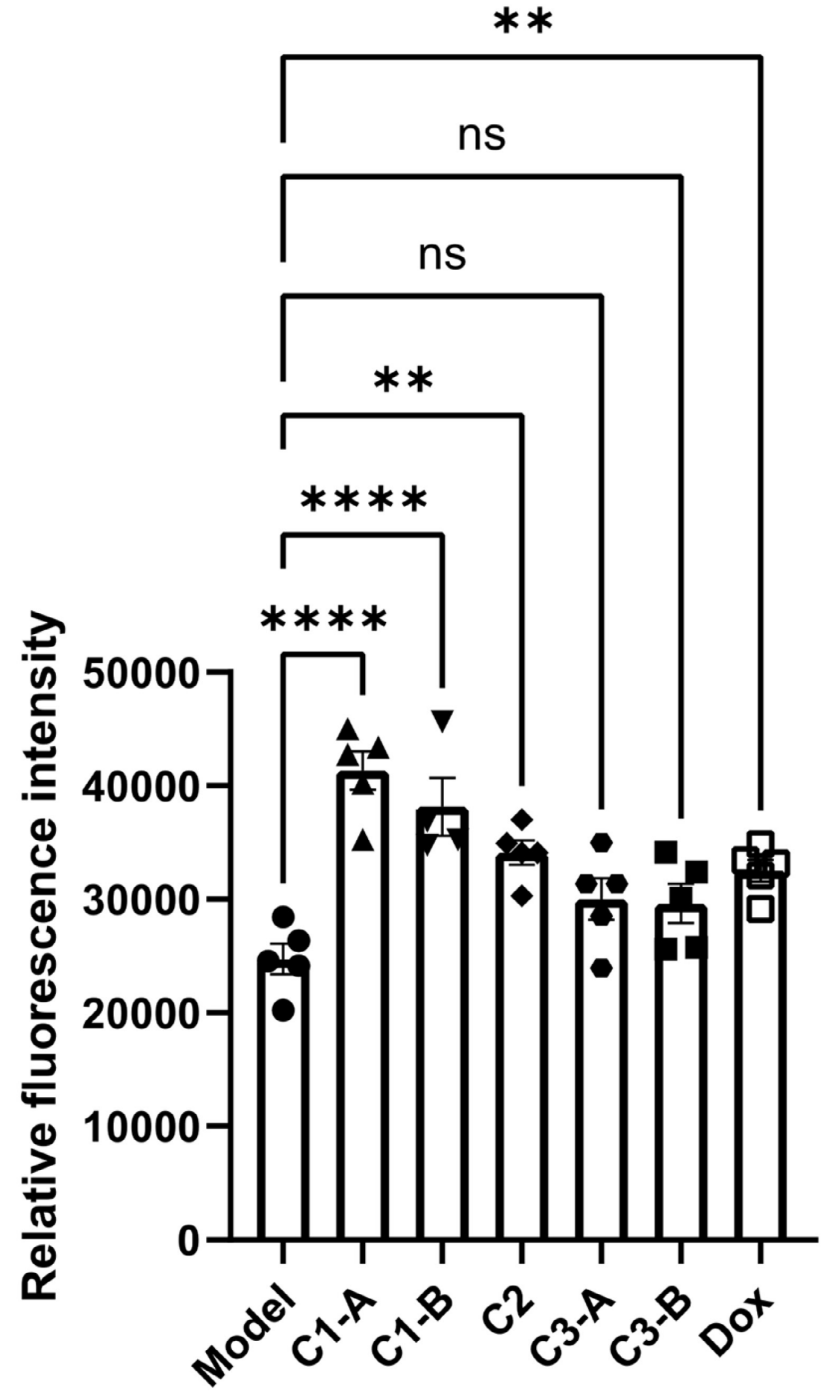
Statistical analysis of DOX IN-NFL@AuNPs (C1-A-C1-B groups) and model groups as reference (Model, DOX, C2; C3-A; C3-B) on the ROS content of PANC-1 cancer cell after i.v. onto mice. Data was shown as Mean ± SD. Data was analyzed by One-way ANOVA, *p<0.05, **p<0.01 and ***p<0.001.

Our studies showed that ROS can oxidatively damage tumor cells and improve tumor cell apoptosis. The experimental results are shown in **Figure 3**, compared with the model group, the C1-A and C1-B groups could significantly increase the ROS content in the tumor cells of mice with pancreatic cancer (p < 0.0001). C1-A group (**DOX IN-NFL@AuNPs** diluted 4 times) have a better effect compared to C1-B group (**DOX IN-NFL@AuNPs** diluted 2 times) and C3-A/C3-B groups corresponding to BIOT-NFL at specific concentrations.

We also estimate that C2 group (**DOX IN PEG AuNPs**) and DOX group (free DOX) both impacts the ROS content in pancreatic cancer cells with similar results. Accordingly of these findings, we can conclude that the combination of DOX and BIOT-NFL at specific doses, confers a better steric and chemical configuration of the complex that improves ROS content in pancreatic cancer cells.

### Blood screening

The number of cells in the whole blood of nude mice was detected using a hemocytometer.

Hematology screening is a relevant analysis to check a good response after chemotherapy treatment in oncology field, and the state of inflammation^50^. As proved previously, several parameters connected to red blood are altered by anthracycline drugs as doxorubicin and other metabolites with consequent alteration of membrane structure and biochemical process of metabolism^51^. Contrarily to chemotherapeutic drugs (i.e. doxorubicin), we demonstrated in previous study that NFL- TBS 60-53 under gold nanoformulation (BIOT-NFL-PEG-AuNPs)^27^ improve the number of WBC, NE and LY (p<0.01), and significantly reduce the content of MCV and MCH (p < 0.01), with an outstanding effect on platelets production^27^. Herein, we discovered that a dual combination of DOX and NFL-TBS 60-53 (BIOT-NFL) into gold formulation did not affect the number of blood cells compared to the model group.

All experimental results are shown in **Figure S2 in Supporting Information.**

### Effect of DOX IN-NFL@AuNPs on IL-6, TNF-α, IFN-γ as pro-inflammatory cytokines

The pro-inflammatory cytokine, plays a central role in oncogenesis, cancer progression, invasiveness, microenvironment changes, treatment resistance and prognosis^52^. Cytokines including IL-6, IFN-γ and TNF-α have been monitored in pre-clinically *in vitro* and *in vivo*, studies playing a key role in immunotherapy and cancer diseases^53^. These cytokines, participate into regulation of immune cell proliferation and the decrease of tumor growth showed in pre-clinical tests *in vitro* and *in vivo*. TNF-α is involved in the host immune response and systemic inflammation^54^, playing a key role in apoptotic cell and tumor necrosis, confirming an excellent anti-tumor activity^55^. IL-6 is a cytokine also implicated in the regulation of several processes of immune system^47^, improving the proliferation of responsive T-cells. TNF-α has been establish to play a key role in anti-tumor activity in order to induce apoptotic cell death and tumor necrosis^56^. In addition, previous studies have demonstrated clear evidence that IFN-γ promoted specific immune responses, through immunological processes on the growth of tumor cells^57^. On the other way, IFN-γ is used as adjuvant for immunotherapy in several types of cancer. Besides, IFN-γ inhibits angiogenesis in tumor tissue, induces regulatory T-cell apoptosis, stimulating the activity of macrophages, confirming a key role in tumor progression^58^.

In the previous study we analyzed a large panel of cytokines with a great improvement onto cytokines by NFL peptide under gold nanoformulation^27^.

**Figure 4:**
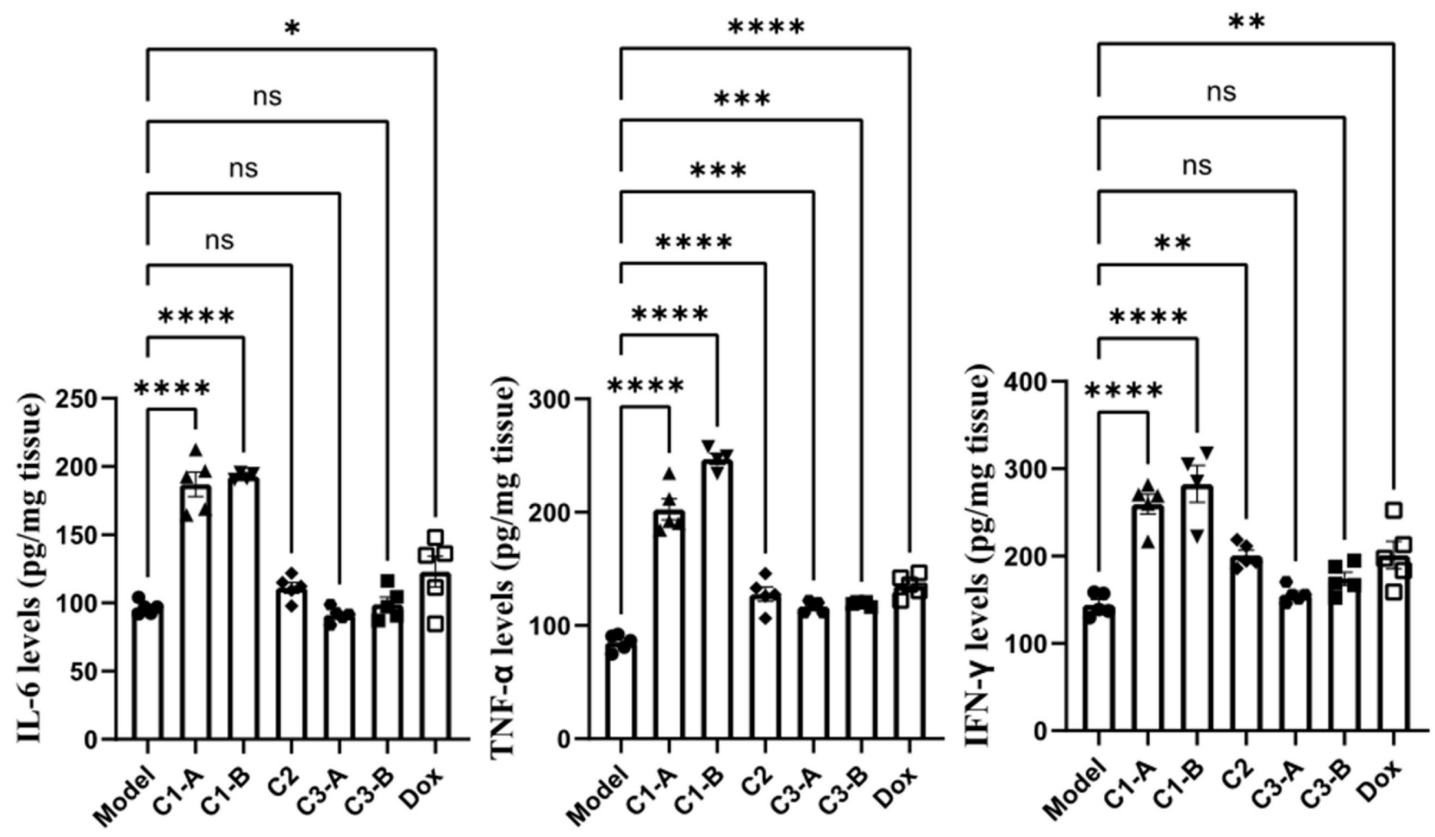
DOX IN-NFL@AuNPs cytokine effect on the content of inflammatory factors in pancreatic cancer mice. Data was shown as Mean ± SD. The data was analyzed by One-way ANOVA, *p<0.05, and **p<0.01.

Herein, we combined and studied a dual effect of DOX and NFL after injection of DOX IN-NFL@AuNPs (C1-A-C1-B groups) onto PDAC tumor-bearing mice, discovering that the levels of serum IL-6, IFN-γ and TNF-α (p<0.05) were strongly improved. Compared to the model group, C1-B can significantly increase the content of TNF α and IL-6 in serum and tumor tissues (p < 0.05); C1-A group can significantly increase the content of IFN γ in serum and tumor tissues (p < 0.05) (**Figure 4**). These results confirm that the different doses of DOX and NFL peptide in the gold nanoformulation influence the biological behavior and the impact on the immunity system.

### Biodistribution and histological evaluation

**Figure 5:**
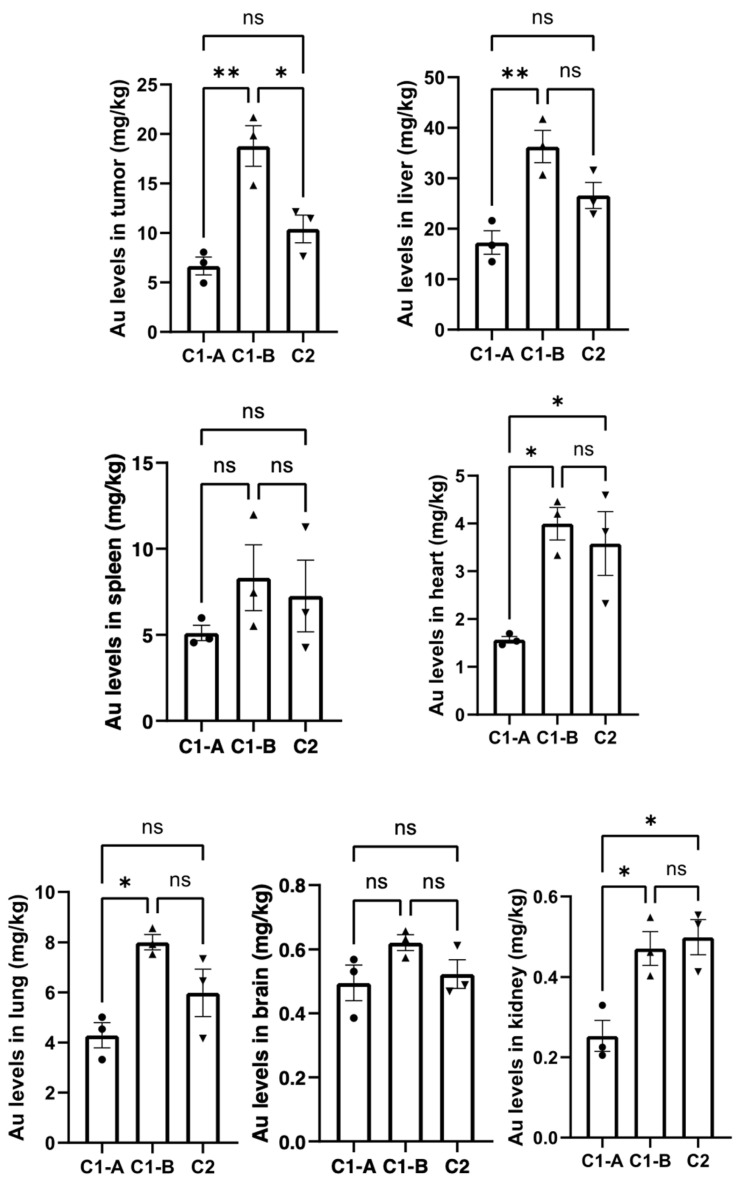
Higher distribution of nanomaterials in tumor tissue of pancreatic cancer mice. Data was shown as Mean ± SD. The data was analyzed by One-way ANOVA, *p<0.05, **p<0.01, ***p <0.001 and ****p<0.0001.

The distribution of gold nanoparticles in each group of organs was detected by ICP-MS after administration^27^, and the experimental results are shown in **Figure 5**. In all tissues, the content of gold nanoparticles in group C1-B was higher than that in groups C1-A and C2.

When nanoparticles are administrated through intravenous system, a large amount of them is stored in the liver. In previous study we showed that the pegylated nanoparticles decorated with NFL-TBS-60-43 (BIOT-NFL) reduce their adsorption in the organs with a strong reduction in the risk of long-term toxicity^27^.

Herein, we have verified the synergic role of DOX and BIOT-NFL in gold nanoformulation after intravenous administration. As showed in **Figure 5**, we observed a great accumulation of DOX IN-NFL@AuNPs at fixed dose (C1-B) in tumor heart and liver. Contrarily, the form C1-A decrease in the liver, due to the presence of DOX at different concentration.

However, the presence of DOX IN-NFL@AuNPs (C1-B) in the lung and kidney confirms the lack of translocation into the circulatory system and secondary organs^59^. We confirmed an excellent accumulation of DOX IN-NFL@AuNPs (C1-B) in the brain that this is crucial to validate the synergic role of our nanovector. These results also suggest that DOX IN-NFL@AuNPs improved lung bioavailability to control excessive inflammation. Furthermore, the rapid excretion of nanoparticles via the kidney could reduce their risk of long-term toxicity. This indicates a reduction in the risk of long-term toxicity.

We can assume that our system DOX IN-NFL@AuNPs (C1-B) showed a good metabolic profile to control the acute inflammation.

**Figure 6:**
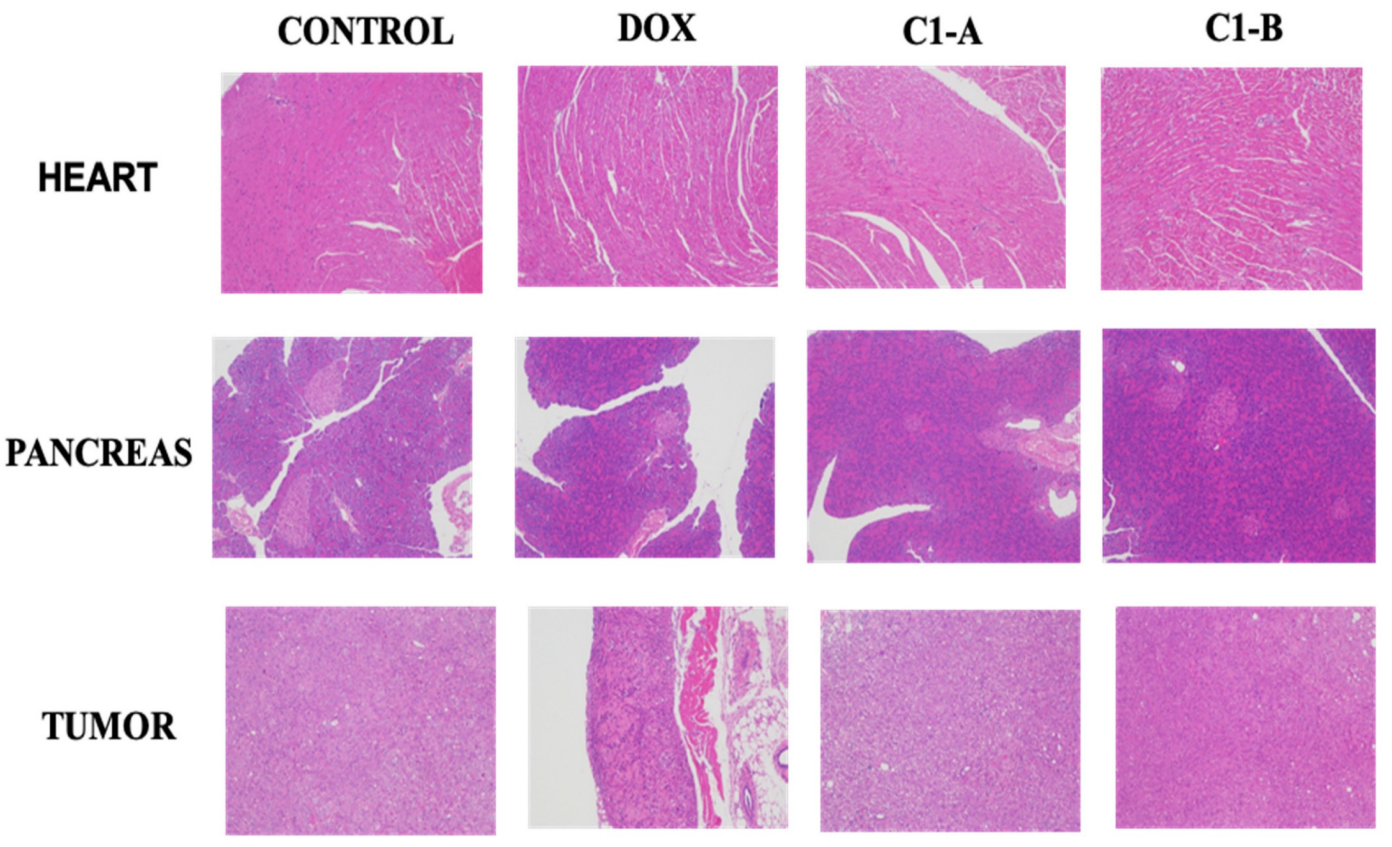
Histological sections of the heart, pancreas and tumor stained by H&E.

As proved previously, DOX is capable to modify iron metabolism triggering ROS production responsible of cardiotoxicity and cardiomyopathy^60^. To evaluate the chronic myocardial toxicity of DOX before and after complexation to nanovector, a group of mice was treated with free DOX and DOX IN-NFL@AuNPs under two forms (C1-A; C1-B) and then analyzed by histology after the last administration. The heart tissue sections from the free DOX group exhibited strong myocardial pathological changes with the presence of characteristic myocardial fibers with several degrees of rupture^28^ (**Figure 6**). After treatment with our nanovector (DOX IN-NFL@AuNPs) (C1-A; C1-B), we observed a disappearance of myocardial fibers confirming the efficacity of our system. In particular we assumed a better result with C1-B, confirming the key role of the dose, chemical-steric arrangement and consequent therapeutic effect. As discussed previously^28^ DOX-gold complex significantly eliminated the chronic myocardial toxicity of DOX during the period of treatment. Indeed, in our methodology, DOX was complexed to BIOT-NFL and gold salt by chelation^30^. In this chemical conformation, DOX does not bind Fe^3+^/Fe^2+^, and does not stimulate the ROS production that is responsible for cardiotoxicity^28^. As illustrated in **Figure 5**, no abnormalities were also observed in the main organs as pancreas and tumor. These results indicated the good tolerance and biosafety of all treatment formulations.

**Scheme 2:**
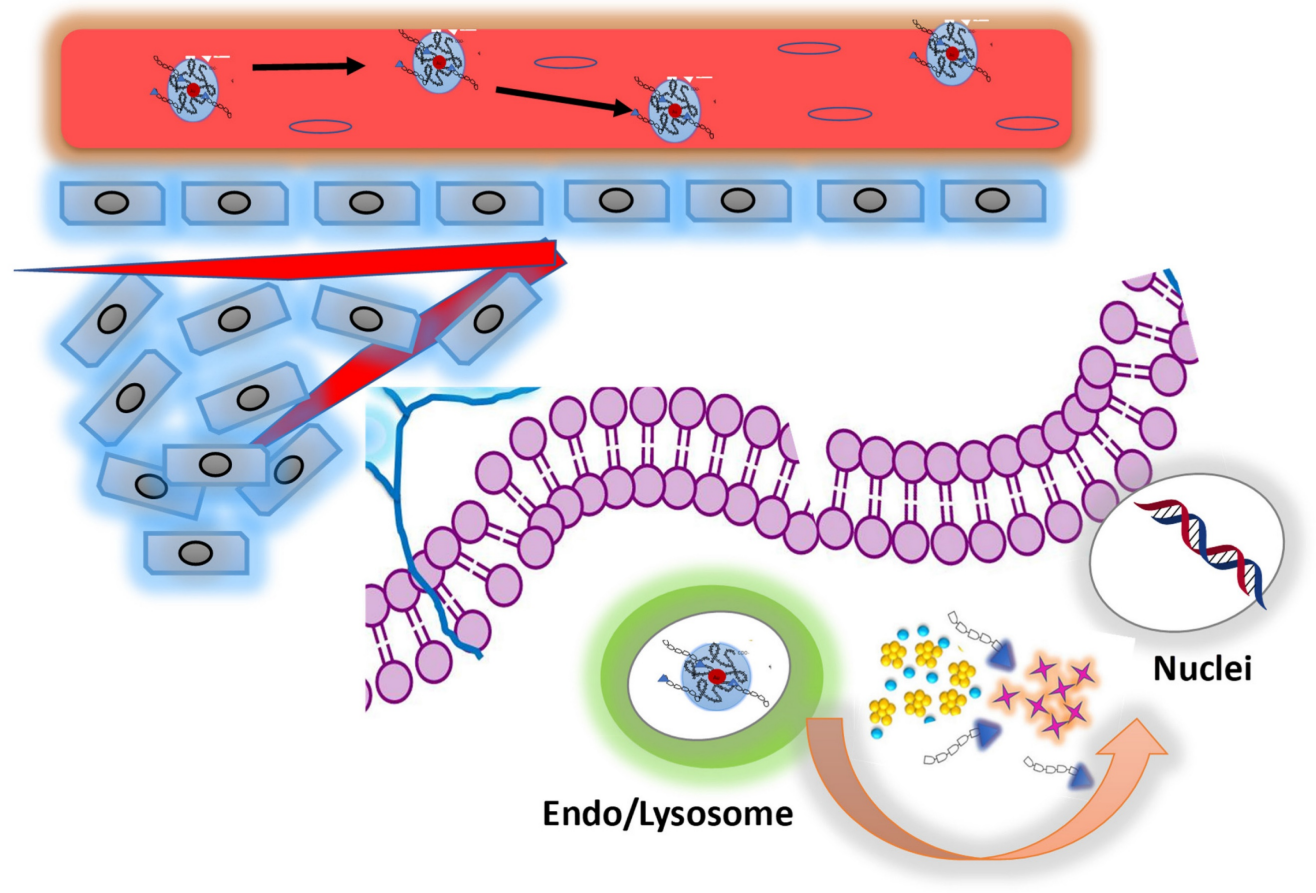
Schematic representation for mechanism of co-delivery of doxorubicin and BIOT-NFL by complexation method for enhanced synergistic cancer therapy.

To evaluate a synergistic action of our nanoformulation, we assume that the combination of DOX and BIOT-NFL, influence the therapeutic effect and biological cellular response (**Scheme 2**). On the basis of our previous studies^27^,^36^, we hypothesized that DOX IN-NFL@AuNPs was stable and could penetrate the cell membrane via lysosomal-mediated pathway^61^.

We think that BIOT-NFL was released, interacting with receptors and inhibiting angiogenesis and tumorigenesis through destroying of microtubules (**Scheme 2**). There is a synergistic effect between DOX and the peptide. Thus, the peptide weakens the cytoskeleton of cells which could improve the effect of DOX. In a related way, DOX released under gold complex, acts on nuclei inhibiting DNA mutated replication^40^. This behavior is very encouraging in order to promote the synergic effect of peptides and chemotherapeutic drugs increasing the immune responses.

## Conclusions

This study reports the potential of DOX IN-NFL@AuNPs to target pancreatic cancer and to improve the synergic anti-tumor efficacy. On the basis of these findings, DOX IN-NFL@AuNPs displayed a significant repression of tumor growth, and higher stimulation of the immune system. Both the data and the new conception of those peptides, present a major synchronized driver to implement the numerical output data provided by the hypothesis computational model, that define the dynamic of the micro-environment of ECM and the multi-physics fluid conditions. Thus, the DOX IN-NFL@AuNPs is promoted to contribute to divert the therapy resistance out of their field of interaction with PDAC, hence, compromising the pro-tumorigenic programs in early stage, or isolate the tumor microenvironment center (TMC) within a sever electromechanical environment. We confirm that the chemical-molecular conception and design plays a key role in the parameters modulating the *in vivo* properties and functionalities of the nano carriers, improving their performance in cancer therapy.

## Materials and Methods

Tetrachloroauric acid (HAuCl_4_*H_2_O), sodium borohydride (NaBH_4_), dicarboxylic PolyEthylene Glycol (PEG)-600 (PEG) (PEG-diacide), phosphate buffered saline (PBS, 0.1 M, pH from 4 to 13), DMEM, Doxorubicin (DOX) (98%), sodium chloride NaCl (0.9%; 99.5%) EDTA, Isoflurane, Paraformaldehyde were purchased by Sigma-Aldrich at maximum purity grade. All solvents were used without any further purification. BIOT-NFL-peptide, was produced by Polypeptide Group (Strasbourg, France). Experiments were carried out at room temperature if not specified otherwise.

### Synthesis of DOX IN-PEG-AuNPs

The synthesis of DOX IN-PEG-AuNPs colloids, used in this study as control, was described previously^21^. Briefly 20 mL of aqueous HAuCl_4_ ( 0.8 mM ) was mixed with 2 mL of DOX at 1 mg/ mL for 1h under stirring at room temperature. After 10 min, 1 mL of PEG diacide at 1mg/mL was added to the solution. Finally, 3 mL of ice-cold 7,93 mM NaBH_4_ was added dropwise.

### Synthesis of BIOT-NFL-PEG-AuNPs

The synthesis of BIOT-NFL-PEG-AuNPs colloids, used in this study as control, was described previously^27^. Briefly 20 mL of HAuCl_4_ aqueous dispersion (0.8 mM) was added to the NFL-peptide solution (0.05 mL, 1 mg mL 1 in water/10% ethanol) and stirred for 20 minutes. Then, 250 mL of PEG diacide (1mM) was added and mixed by magnetic stirring at room temperature. Finally, 1.8 mL of NaBH4 (3 mg/10 mL) was added at once. The formation of the BIOT-NFL-PEG-AuNPs was indicated by an instantaneous color change of the dispersion from pale yellow to bright pink-purple after the addition of the reducing agent. The “as-prepared” BIOT-NFL-PEG-AuNP dispersion was purified by centrifugation three times at 9 000 rpm for 9 minutes; then, the supernatant was discarded.

### Synthesis of DOX IN-NFL@AuNPs

20 ml HAuCl_4_ aqueous solution (2.5×10^-4^ M) was added to 100µl of NFL-DOX solution and was stirred for 30 min. After 30 min, 250 µl of dicarboxylic PEG was added and mixed by magnetic stirring for 10 min at room temperature. Finally, 1.2 ml of aqueous 0.01 M NaBH_4_ was added at once. The formation of the DOX IN-NFL@AuNPs was confirmed by a color change of the solution after reduction (NaBH_4_) and proved by UV-VIS and Raman Spectroscopy (data not show).

Products of each synthetic step were stored at 27-29°C and characterized by UV-Vis spectroscopy and Transmission Electron Microscopy (TEM). The “as-prepared” DOX IN-NFL@AuNPs solution was centrifuged at 6,000 rpm for 10 min for three times; then, the supernatant was discarded. This was repeated twice to remove excess of not-conjugated dicarboxylic PEG.

### Preparation of DOX-NFL (DOX-NFL)

The BIOT-NFL in powder was diluted in water and ethanol (100/900µl) at 1.8 nM of concentration. A DOX solution was prepared at 17µM of concentration. 200 µl de BIOT NFL was then diluted to 980 µl of DOX.

### Physicochemical evaluation

All the measurements were performed in triplicate in order to validate the reproducibility of the synthetic and analytical procedures. All the measurements were carried out as previously described^21^.

### UV/Vis measurements

The absorption spectra of all NPs were recorded in water at a concentration of 10^-4^ M. All spectra were recorded using a Perkin Elmer Lambda UV/Vis 950 spectrophotometer in plastic cuvettes with an optical path of 10 mm. The wavelength range was 200-900 nm.

### Transmission Electron Microscopy (TEM)

All microscopy analyses were realized as previously described^21^. Briefly 2 mL of each sample was deposited on copper grids (150 mesh) and stained with 2% uranyl acetate for one minute, and then each sample was dried at room temperature before observation. The examination was performed usinga120 kV Jeol JEM-1400 electron microscope (Jeol, Japan) equipped with a Gatan SC1000 ORIUS® CCD camera (11 Megapixel) from the USA.

### Dynamic light scattering (DLS)

The size measurements were performed using a Zetasizer Nano ZS (Malvern Instruments, Malvern, UK) equipped with a He-Ne laser (633 nm, fixed scattering angle of 173°) at room temperature.

### Zeta potential measurements

All measurements were carried out as previously described^21^.

### Loading and release

All experiments about drug/peptide loading and release were carried out as previously described^19^,^21^.

## Supplementary Material

Supplementary methods, figures and tables.

## Figures and Tables

**Table 1:** Animal grouping and drug administration.

Group	Drug	Quantity	Administration of injection	Administration frequences
C1-A	DOX IN-NFL@AuNPs working solution diluated 4 times	5	i.v. 1.25 mg/ml	3/week
C1-B	DOX IN-NFL@AuNPs working solution diluated 2 times	5	i.v. 1.25 mg/ml	3/week
C2	DOX-IN working solution diluated 2 times	5	i.v. 2.5 mg/ml	3/week
C3-A	NFL (Equal dose to group C1-A)	5	i.v.	3/week
C3-B	NFL (Equal dose to group C1-B)	5	i.v.	3/week
DOX	DOX	5	i.v.	3/week
