# Supplementary material for: Doxorubicin-NFL-TBS.40-63 peptide Gold Complex Nanovector (DOX IN-NFL@AuNPs): Efficacy Evaluation in a mouse transplantation tumor model induced by PANC-1/ADR human pancreatic cancer resistant strain cells
Source: Nanotheranostics. 2025 Jun 19;9(2):186–98. doi: 10.7150/ntno.109280 (PMC12188537; doi:10.7150/ntno.109280)
Supplement: Supplementary file 1 — Supplementary methods, figures and tables. [file ntnov09p0186s1.pdf]

Supporting Information

Doxorubicin-NFL-TBS.40-63 peptide Gold Complex Nanovector (DOX IN-NFL@AuNPs): Efficacy Evaluation in a mouse transplantation tumor model induced by PANC-1/ADR human pancreatic cancer resistant strain cells

Hui Liu[1 ‡], Qiqian Liu[2‡], Xiaowu Li[1], Abdelkader Boucetta[Z], Joel Eyer[3],

Jolanda Spadavecchia[Z]*

[1] Department of Hepatobiliary Surgery, Guangdong Provincial Key Laboratory of Regional Immunity and Diseases & Carson International Cancer Center， Shenzhen University General Hospital & Shenzhen University Clinical Medical Academy Center, Shenzhen University, Shenzhen, 518083 China.

[2] CNRS, UMR 7244, NBD-CSPBAT, Laboratoire de Chimie, Structures et Propriétés de Biomatériaux et d'Agents Thérapeutiques Université Paris 13, Sorbonne Paris Nord, Bobigny 93000, France; University Sorbonne Paris Nord; CB3S, UMR CNRS 7244, Insitut Galilée, 99 France.

[3] Laboratoire Micro et Nanomédecines Translationnelles, Inserm 1066, CNRS 6021, Institut de Recherche en Ingénierie de la Santé, Bâtiment IBS Institut de Biologie de la Santé, Université d'Angers, Centre Hospitalier Universitaire, Angers, 49100 France.

Corresponding Authors*: Jolanda Spadavecchia (jolanda.spadavecchia@univ-paris13.fr; jolanda.spadavecchia@gmail.com)

DLS /PZ analysis of DOX IN-NFL@AuNPs

Results

			Size (d.nm...	% Intensity:	St Dev (d.n...
Z-Average (d.nm):	85,90	Peak 1:	109,2	100,0	41,98
PdI:	0,214	Peak 2:	0,000	0,0	0,000
Intercept:	0,896	Peak 3:	0,000	0,0	0,000
Result quality :	Good				

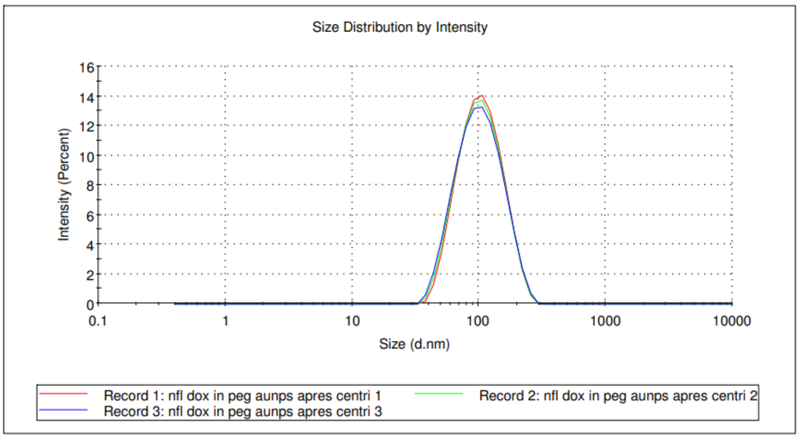

Results

			Mean (mV)	Area (%)	St Dev (mV)
Zeta Potential (mV):	-35,7	Peak 1:	-34,8	65,2	6,22
Zeta Deviation (mV):	12,0	Peak 2:	-20,6	18,5	4,13
Conductivity (mS/cm):	0,390	Peak 3:	-55,6	16,3	6,65
Result quality	See result quality report				

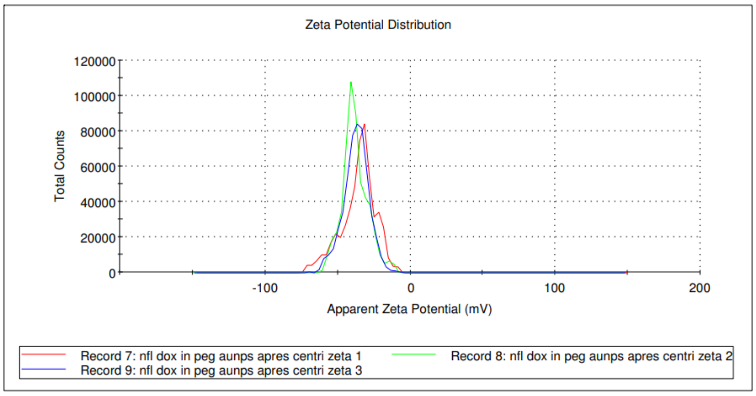

Animal information

Laboratory Animals and Breeding Management (See Attachment 2 for details of animal qualification certificate)

Species:Rodent; Lineage:BALB/c-nude; Animal Class:SPF; Sex:Female; Weight:20-22 g; No. of Mice:37.

Animal Production License No.: SCXK(GD)2023-0059, Guangzhou Rigel Biotechnology Co.

Animal Certificate No.: No.44827200006654 and No.44827200006856.

Laboratory Use License No.: SYXK(GD)2023-0259, Guangzhou Rigor Biotechnology Co.

Animals were kept in accordance with the SPF Regulations for Laboratory Animal Husbandry of Guangzhou Rigel Biotechnology Co., Ltd. with the room temperature maintained at 20-26 °C, humidity

at 40-70%, and the room maintained at a 12h day/night rhythm, and the mice were allowed to eat and drink freely, so that they could adapt to the new environment for at least 3 days, and all the operations were conducted in accordance with the regulations of the Ethics Committee for Laboratory Animals of Guangzhou Rigel Biotechnology Co.

Experimental Methods

PANC-1/ADR cell culture

PANC-1/ADR cells were cultured in DMEM high glucose medium containing 10% fetal bovine serum included 1% P/S and 1 µg/mL Dox. the cells in logarithmic growth phase were used for the experiment and the incubator was set at 5% CO_2 concentration and 37 °C.

Animal quarantine

After the mice enter into the SPF laboratory center, the mice will be quarantined in strict condition with the relevant technical requirements of the SPF laboratory center of Guangzhou Ruige Biotechnology Co.

Inspection of animal: weigh the mice before and after entering in the center ; check the body and the shape of the mice, coordination of movement, respiration, and all the physics of the mices, at the end a microbiological testing of mice has been carried out for determine the follow-up microbiological testing according to the latest quarantine time in the Laboratory Animal Certificate number issued by the purchasing organization.

Animal grouping and drug administration (see Table 1)

Remarks.

C1, C2 reagents, DOX concentration is 1 mg/ml,

The NFL concentration in C1 is 36.8 µmol/ml.

In C3, 2 mg of NFL was dissolved in 100 µl of anhydrous ethanol, and 1900 µl of sterile water was added to obtain a concentration of 368 µmol/ml (master batch), which was then diluted 10-fold to obtain 36.8 µmol/ml of NFL.

For a 50 µl/10 g injection, the DOX concentration should be 0.5 mg/ml for a 2.5 mg/kg injection. The current concentration of DOX in both C1 and C2 is 1 mg/ml, so a 2-fold dilution is required to begin use.

Currently, the concentration of DOX in C1 and C2 is 1 mg/ml, so a 2-fold dilution is needed to start the use of D.X

C3-A group: C1 working solution was diluted 4 times and injected at 50 µl/10 g. At this time, the concentration of C1 was 9.2 µmol/ml, and the C3 working solution was diluted 4 times and injected.

C3-B group: the working solution was diluted 2 times, injected according to 50 µl/10 g, at this time, the concentration of C1 was 18.4 µmol/ml, and the C3 working solution was diluted 2 times and injected.

Molding method

PANC-1/ADR human pancreatic cancer resistant cells were digested and counted, the cell density was adjusted to 6×10^7 cells/mL, and then mixed with melted Matrigel matrix gel in equal volume (cell density of 3×10^7 cells/mL), after the preparation of celles,all the mice were anesthetized with isoflurane, injected subcutaneously into the anterior axillae with 0.1 mL of cell suspension (the number of inoculated cells was 3×10^6 cells), During the growth of the tumors and the length (mm) and width (mm)was measured every 3 days starting from day 4 (DIV4). and the first day of inoculation of the tumor cells was recorded as DIV1, the drug was administered for 2 weeks consecutively.

Experimental indicators

Observation of general conditions and weighing of mice

The general condition of the mice was observed veryday. The mice were weighed every 3days After the death of mice after 28 days , tumors, heart, liver, spleen, lungs and kidneys, were sperated , weighted and photographed . Tumors and organs coefficient were calculated with the following equation :

tumor (organ) coefficient = tumor (organ) weight (g)/nude mouse body weight (g).

Tumor Volume Detection

Tumor length (mm) and width (mm) were measured by using vernier calipers at 3 d intervals after tumor cell inoculation with following equation

Tumor volume = π/6*length*width 2.

Histological analysis

Tumors were excised from mice, weighed with an electronic balance, and immediately placed into 12 specimens of liquid nitrogen at -196 °C for 2-3 h,then transferred to a refrigerator at -80 °C for storage. Some of the tumors were fixed in 4% paraformaldehyde and embedded in paraffin for section, after the sections, they were stained with H&E to observe the pathological properties by microscope.

Blood test

At the endpoint of the experiment, blood was taken from the eye socket and placed in a tube containing dipotassium EDTA, mixed and left for 15-20 min.

The test was completed within 30 min to 2 h in the machine. Before the test, a blank was made.The indicators include white blood cell (WBC), neutrophil (NE), lymphocyte (LY), monocyte (MO), eosinophil (EO), basophil (BA), neutrophil ratio (NE%), lymphocyte ratio (LY%), monocyte ratio (MO%), eosinophil ratio (EO%), basophil ratio (BA%), Red blood cells (RBC), hemoglobin (HGB), red blood cell pressure (HCT), mean corpuscular volume (MCV), mean hemoglobin volume (MCH), mean corpuscular concentration (MCHC), red blood cell distribution width (RDW), platelets (PLT), platelet corpuscle (PCT), mean platelet corpuscular volume (MPV), mean platelet distribution width (MDW).

Tissue(1) and ELISA assays (2) Serum collection

the experiment has gone through 2 processing , tissue processing and Elisa assays, first the blood was collected from the abdominal aorta of mice, stock at 4 °C for 2-3 h. Then centrifuged at 3500 rpm for 10 min at 4 °C, the supernatant was separated and stored at -80 °C.

(1) Tissue processing

Tumor tissues were homogenized and centrifuged according to the instructions of the kit, and the protein concentration in the supernatant was measured according to the BCA protein quantification method, the inflammatory factor content in the tissues was measured according to the ELISA method, and the relative content was calculated as sample concentration (pg/mL)/protein concentration (mg/mL).

(2) ELISA assay

All reagents and components were brought to room temperature for the assay with indicated protcole.

Determination of Reactive Oxygen Species in Subcutaneous Tumor Tissue

A single cell suspension came from tumor tissues was prepared by using Meitianni Mouse Tumor Kit. After cell counting, DCFH-DA fluorescent probe (concentration of 5 μmol/L) was added and incubated at 37°C for 30 min. The cells were washed with PBS for three times, and then resuspended with PBS ,the intensity of the fluorescence was measured by a multifunctional enzyme labeling instrument.

CD31 immunohistochemical staining

Dewaxing and rehydration of the tumor tissues , an immunohistochemcal staining has been gone through the standard proctoles: baking, antigen repair, sealing, CD31 staining, Secondary antiboday incubation and photographing

Biodistribution (ICP-MS) Detection

The weighed samples were integrated into a 50 mL digestion vessel tube, 6-8 ml of aqua regia and 1 ml of hydrogen peroxide were added in the tube , at the end a 16 mL graphite heating plate was prepared for 60-150 min digestion at 120-200 ºC.

Statistical Methods

The data were plotted using GraphPad Prism 10.0 statistical software, and the data were plotted using the mean ± standard error (mean± s.e.m.), and the differences of data between different groups were analyzed by one-way ANOVA or two-way ANOVA, and then Tukey's test or Duncan's test was used to indicate significant differences by $P < 0.05$. statistically significant difference.

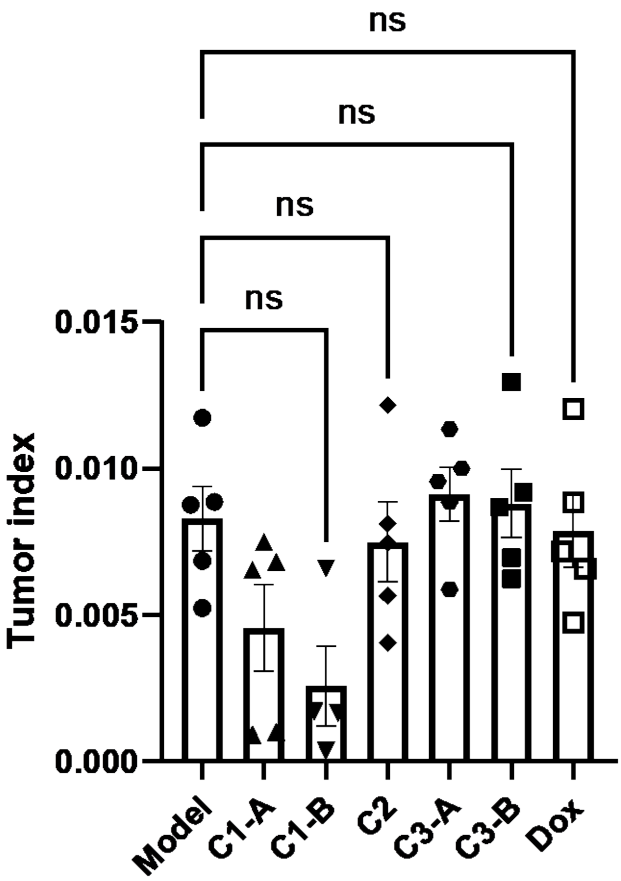
ns
ns
ns
ns
Tumor index
0.015
0.010
0.005
0.000
Model
C1-A
C1-B
C2
C3-A
C3-B
Dox

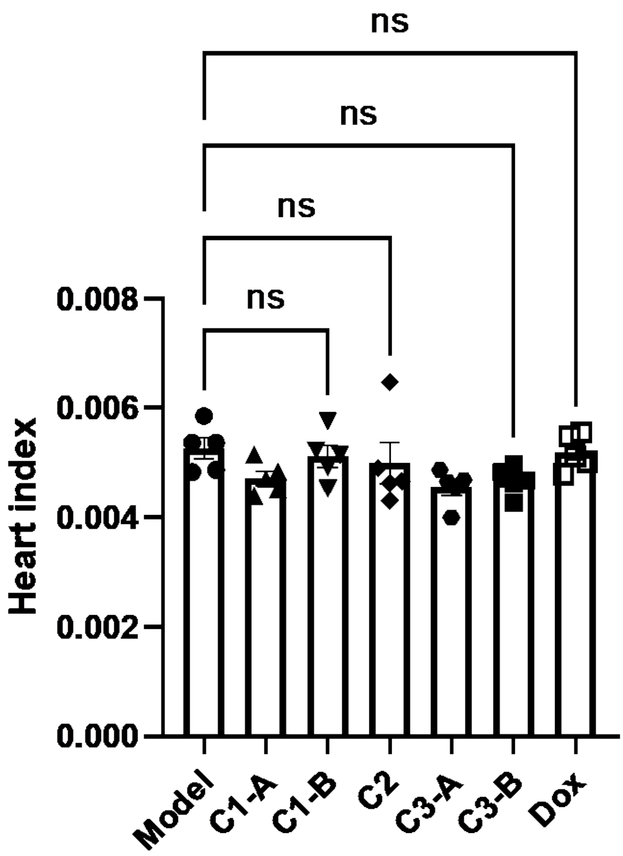
ns
ns
ns
ns
Heart index
0.008
0.006
0.004
0.002
0.000
Model
C1-A
C1-B
C2
C3-A
C3-B
Dox

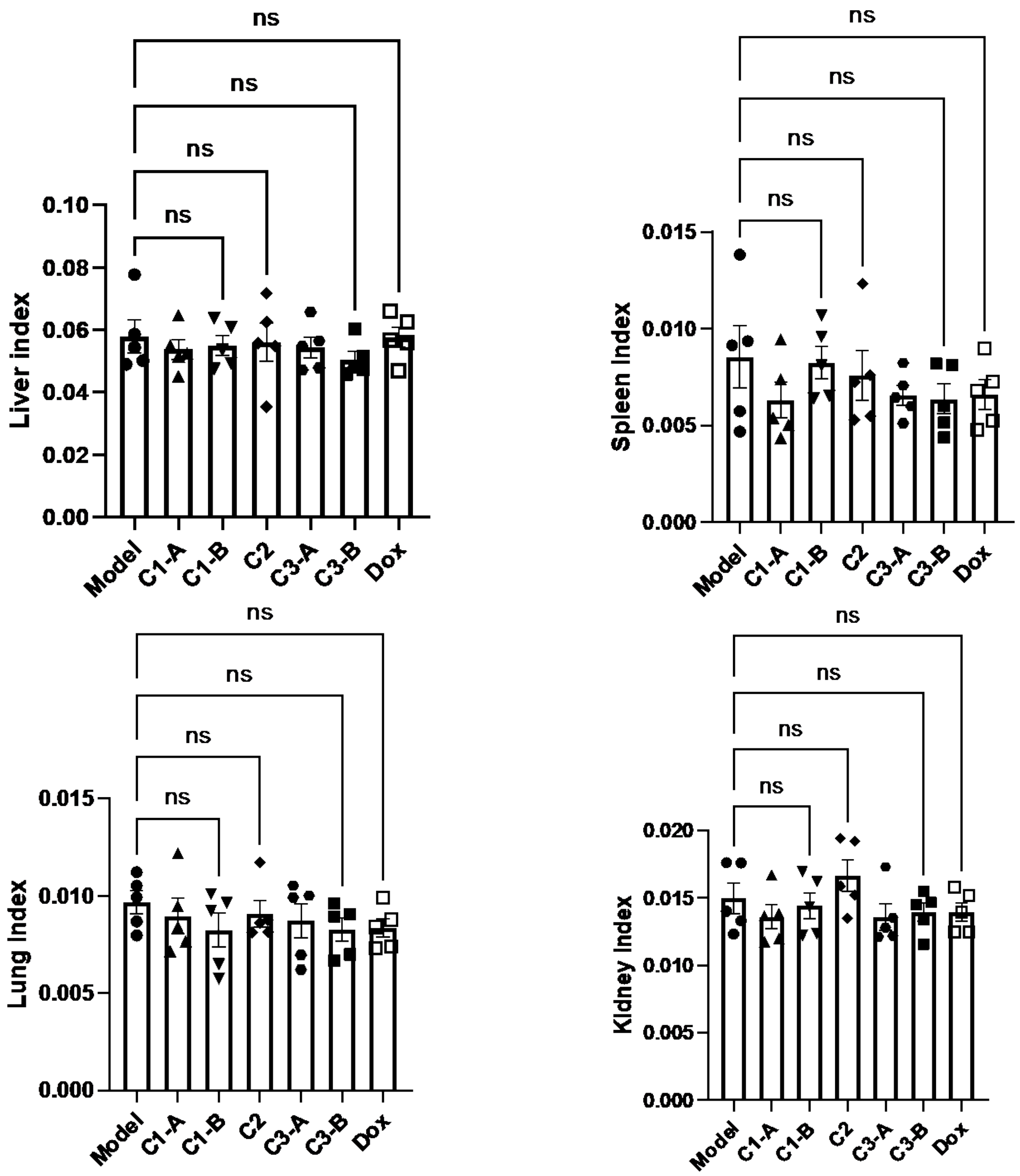
ns
ns
ns
ns
Liver index
0.10
0.08
0.06
0.04
0.02
0.00
Model
C1-A
C1-B
C2
C3-A
C3-B
Dox
Spleen Index
0.015
0.010
0.005
0.000
Lung Index
Kidney Index
0.020

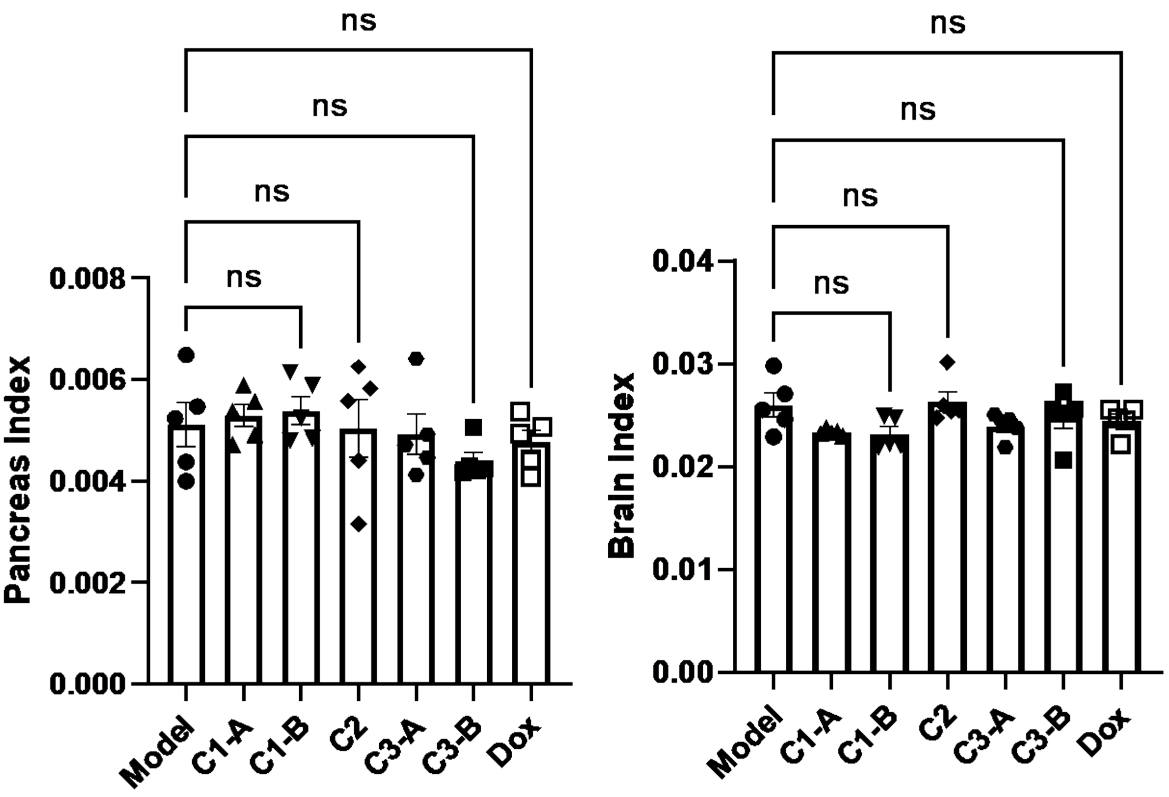

Figure S1: Tumor indices, in all organs for each group.

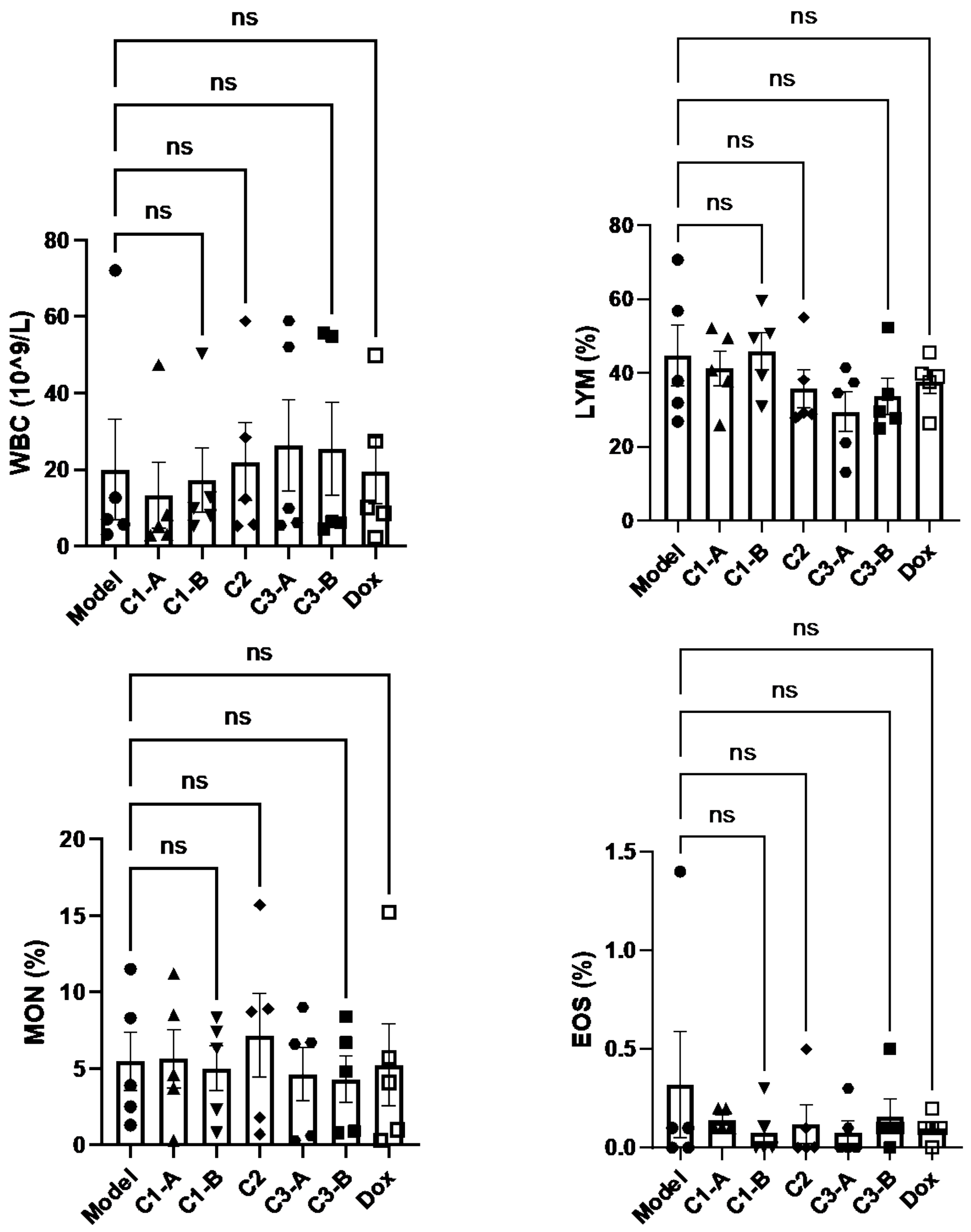
ns
WBC (10^9/L)
0
20
40
60
80
Model
C1-A
C1-B
C2
C3-A
C3-B
Dox
LYM (%)
MON (%)
0
5
10
15
20
EOS (%)
0.0
0.5
1.0
1.5

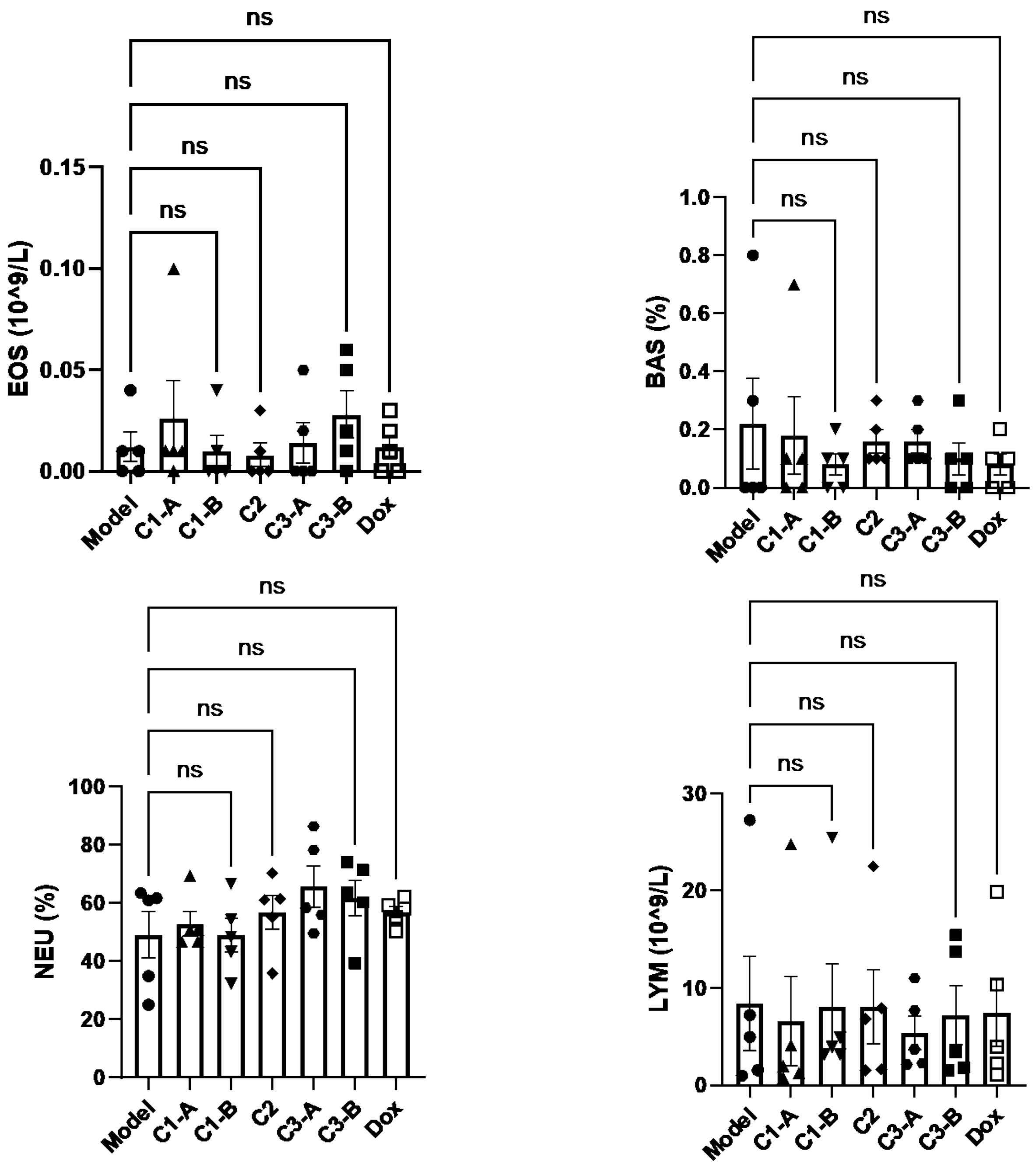
ns
EOS (10^9/L)
0.15
0.10
0.05
0.00
Model
C1-A
C1-B
C2
C3-A
C3-B
Dox
BAS (%)
1.0
0.8
0.6
0.4
0.2
0.0
NEU (%)
100
80
60
40
20
0
LYM (10^9/L)
30
20
10
0

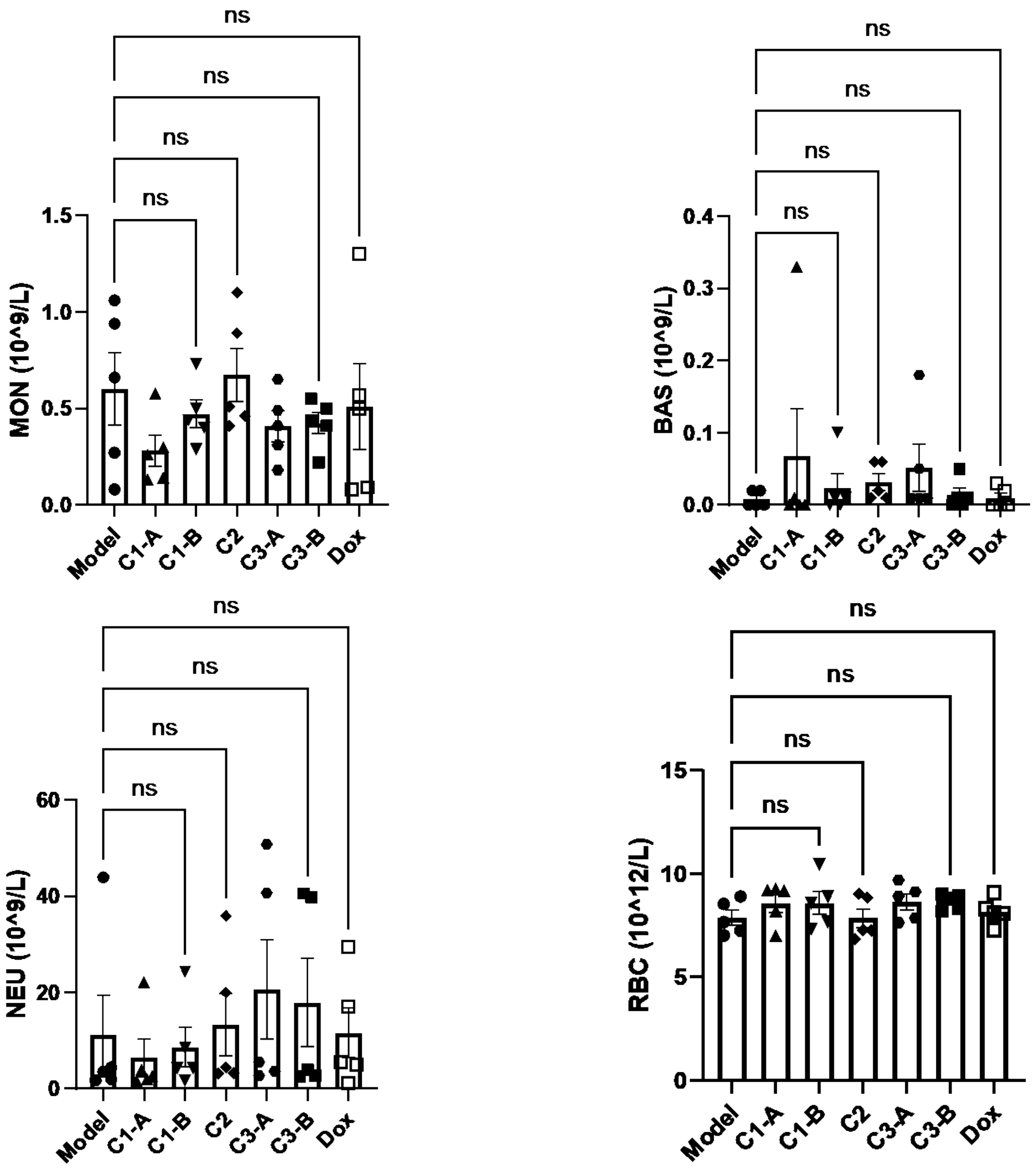

MON (10^9/L)
BAS (10^9/L)
NEU (10^9/L)
RBC (10^12/L)
ns
Model
C1-A
C1-B
C2
C3-A
C3-B
Dox

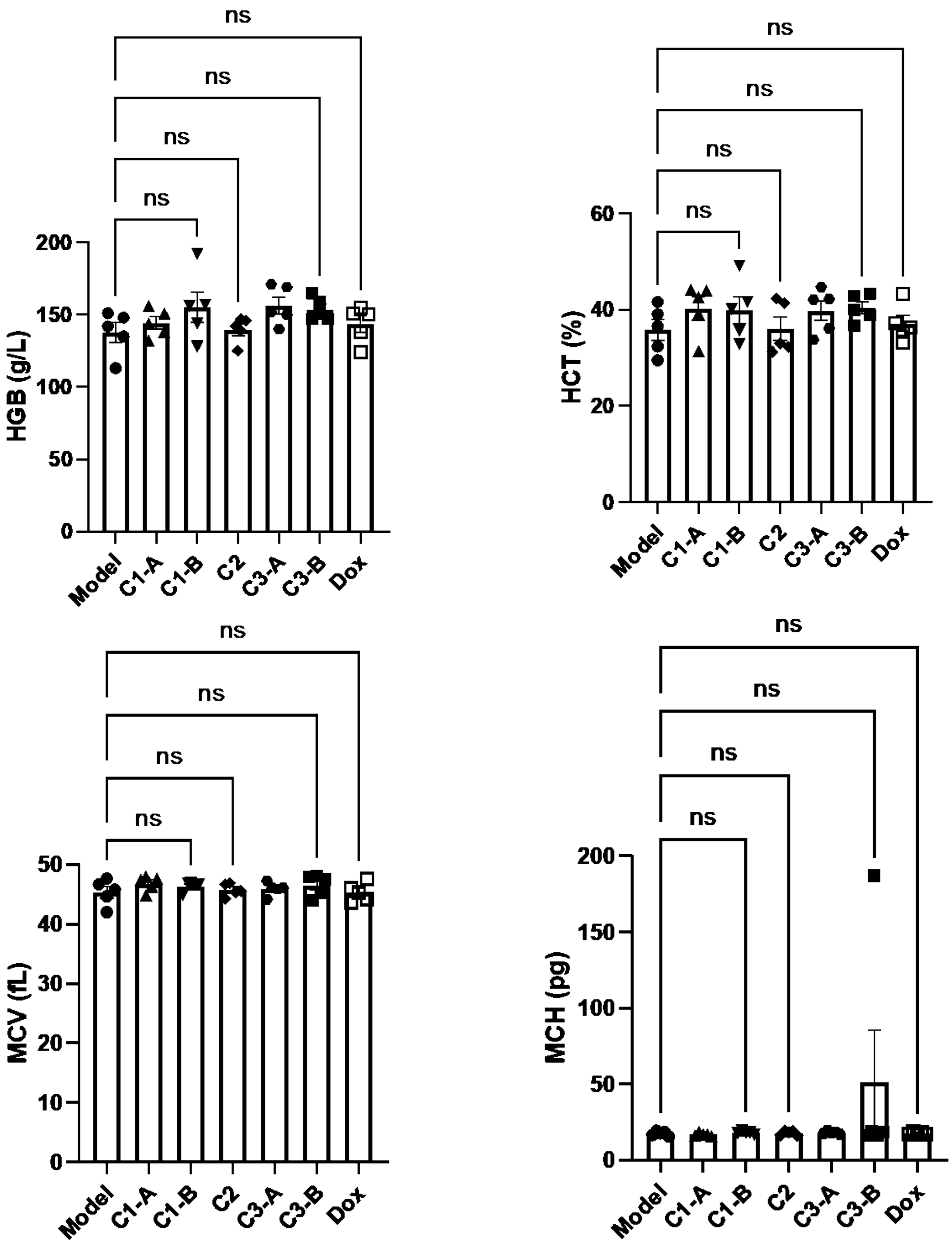
ns
HGB (g/L)
0
50
100
150
200
Model
C1-A
C1-B
C2
C3-A
C3-B
Dox
HCT (%)
0
20
40
60
MCV (fL)
0
10
20
30
40
50
MCH (pg)
0
50
100
150
200

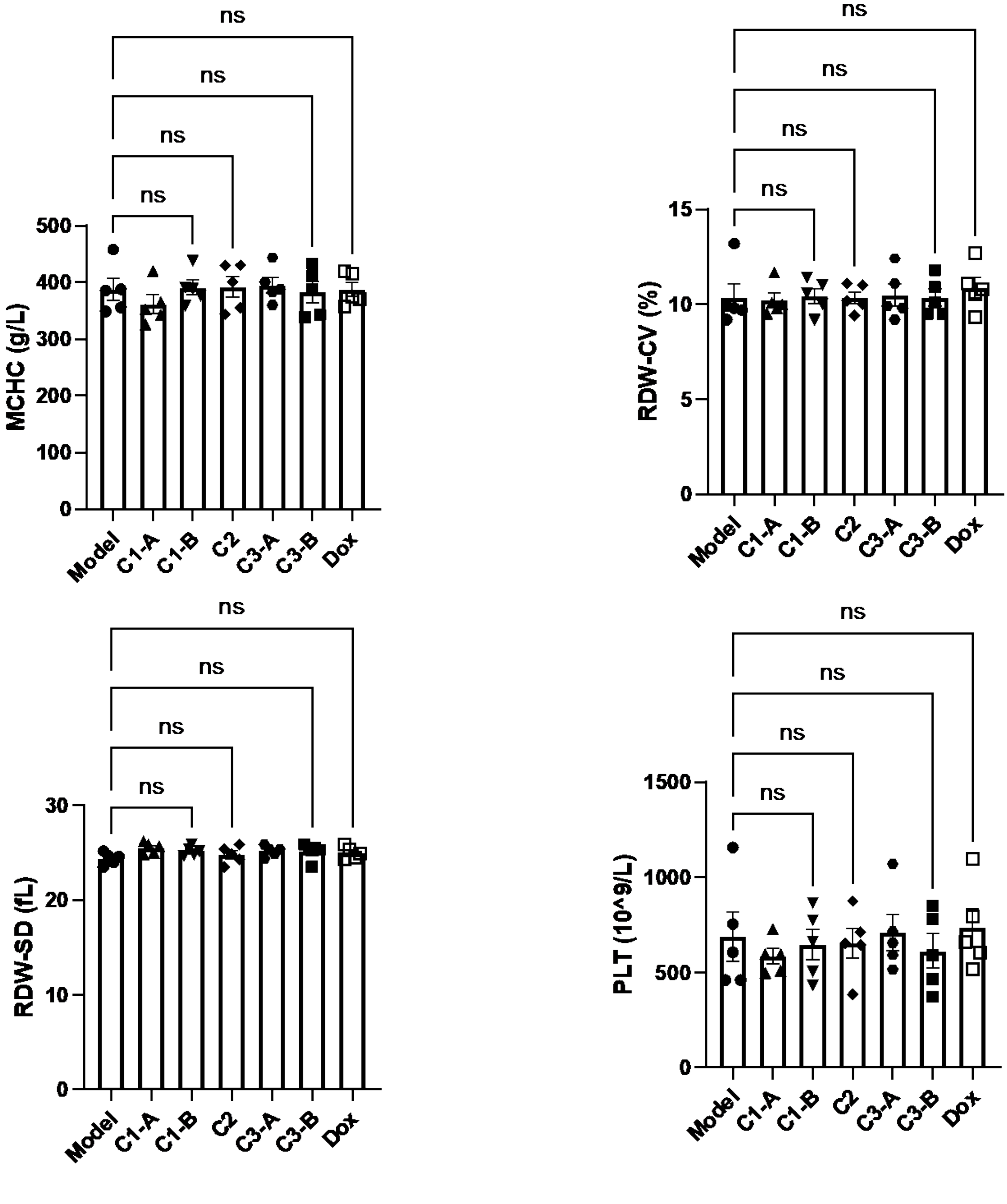
MCHC (g/L)
0
100
200
300
400
500
Model
C1-A
C1-B
C2
C3-A
C3-B
Dox
ns
ns
ns
ns
RDW-CV (%)
0
5
10
15
Model
C1-A
C1-B
C2
C3-A
C3-B
Dox
ns
ns
ns
ns
RDW-SD (fL)
0
10
20
30
Model
C1-A
C1-B
C2
C3-A
C3-B
Dox
ns
ns
ns
ns
PLT (10^9/L)
0
500
1000
1500
Model
C1-A
C1-B
C2
C3-A
C3-B
Dox
ns
ns
ns
ns

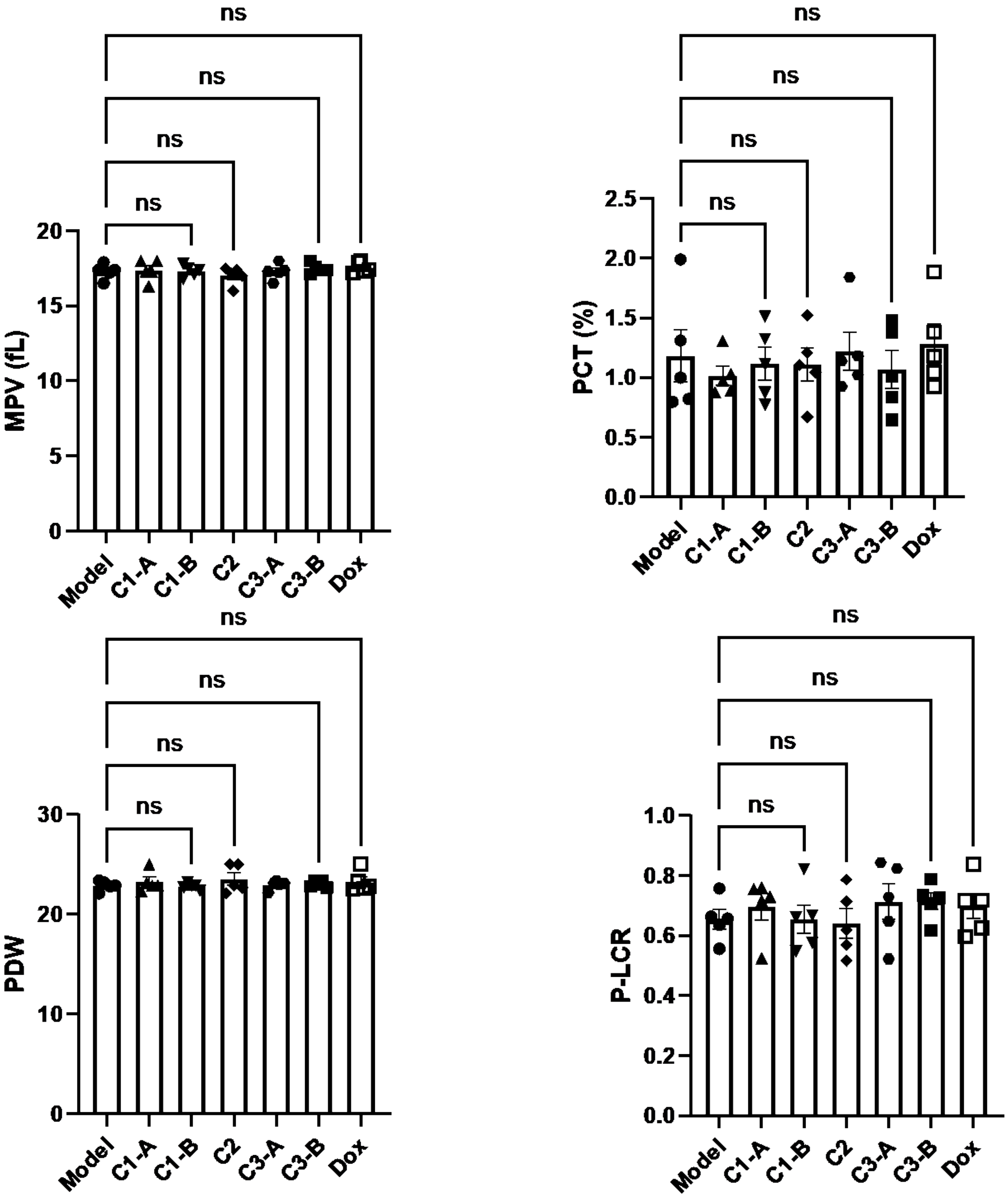
ns
ns
ns
ns
MPV (fL)
0
5
10
15
20
Model
C1-A
C1-B
C2
C3-A
C3-B
Dox
PCT (%)
0.0
0.5
1.0
1.5
2.0
2.5
PDW
0
10
20
30
P-LCR
0.0
0.2
0.4
0.6
0.8
1.0

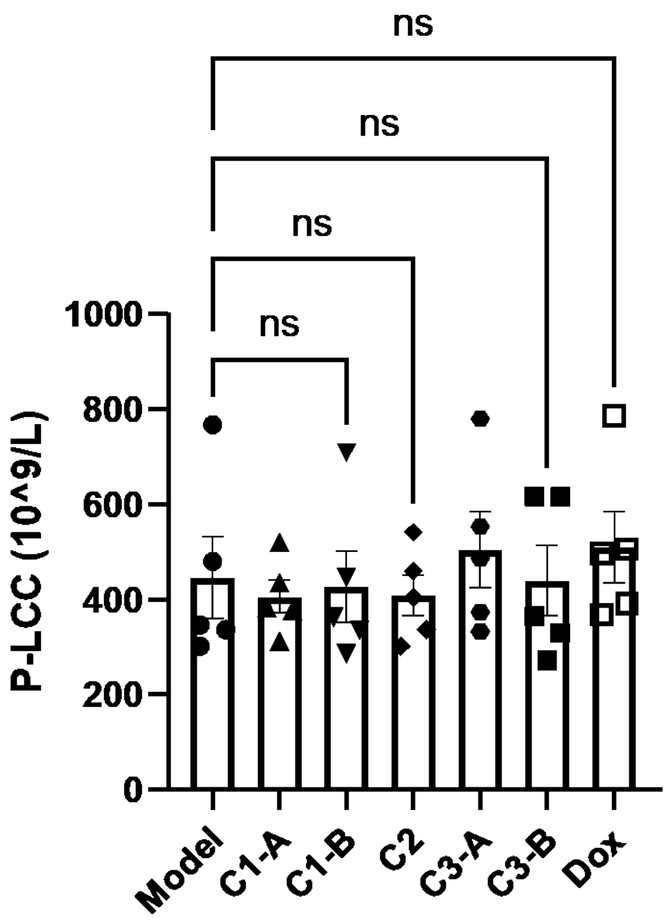

Figure S2: Hematology screening of blood cells by hemocytometer.
